# Prevention and Treatment of Acute Myeloid Leukemia Relapse after Hematopoietic Stem Cell Transplantation: The State of the Art and Future Perspectives

**DOI:** 10.3390/jcm11010253

**Published:** 2022-01-04

**Authors:** Salvatore Leotta, Annalisa Condorelli, Roberta Sciortino, Giulio Antonio Milone, Claudia Bellofiore, Bruno Garibaldi, Giovanni Schininà, Andrea Spadaro, Alessandra Cupri, Giuseppe Milone

**Affiliations:** Division of Hematology, AOU “Policlinico G. Rodolico-San Marco”, Via Santa Sofia 78, 95124 Catania, Italy; leotta3@yahoo.it (S.L.); roberta_sciortino@virgilio.it (R.S.); giulio.milone89@gmail.com (G.A.M.); bellofioreclaudia@gmail.com (C.B.); brunga93@gmail.com (B.G.); giovanni.schin@gmail.com (G.S.); andrea.spadaro@hotmail.it (A.S.); alessandracupri@hotmail.it (A.C.); giuseppe.milone@gmail.com (G.M.)

**Keywords:** allogeneic bone marrow transplantation, relapse, acute leukemia

## Abstract

Allogeneic hematopoietic stem cell transplantation (HSCT) for high-risk acute myeloid leukemia (AML) represents the only curative option. Progress has been made in the last two decades in the pre-transplant induction therapies, supportive care, selection of donors and conditioning regimens that allowed to extend the HSCT to a larger number of patients, including those aged over 65 years and/or lacking an HLA-identical donor. Furthermore, improvements in the prophylaxis of the graft-versus-host disease and of infection have dramatically reduced transplant-related mortality. The relapse of AML remains the major reason for transplant failure affecting almost 40–50% of the patients. From 10 to 15 years ago to date, treatment options for AML relapsing after HSCT were limited to conventional cytotoxic chemotherapy and donor leukocyte infusions (DLI). Nowadays, novel agents and targeted therapies have enriched the therapeutic landscape. Moreover, very recently, the therapeutic landscape has been enriched by manipulated cellular products (CAR-T, CAR-CIK, CAR-NK). In light of these new perspectives, careful monitoring of minimal-residual disease (MRD) and prompt application of pre-emptive strategies in the post-transplant setting have become imperative. Herein, we review the current state of the art on monitoring, prevention and treatment of relapse of AML after HSCT with particular attention on novel agents and future directions.

## 1. Introduction

Relapse of AML after HSCT affects about 30–40% of HSCT recipients [1,2,3]. The prognosis of relapse of AML after HSCT is poor with a 3-year survival rate of around 20–30% [1,2]. Thanarajasingham et al. by analyzing the outcome of the patients who relapsed after HSCT at the Dana–Farber Cancer Institute between 2004 and 2008 have found that early relapse (<6 months from HSCT), relapse after a myeloablative conditioning, previous graft-versus-host disease (GVHD), HCT-CI ≥ 3 and disease-risk index high/very high were associated with worse outcome. Based on these variables a prognostic model was calculated with a 3-year survival rate ranging from 36% for low risk (<3 high-risk variables) to 3% for high-risk patients (≥5 variables) [2]. Bejanyan et al. [4] analyzed data from the CIBMTR database of 1788 patients affected by AML relapsing after HSCT between 1990 and 2010. Three-year survival ranged from 4% for patients who relapsed within 6 months to 38% for those relapsing later than 3 years from HSCT. In the multivariate analysis, factors associated with better survival were the time to relapse after HSCT and the previous reduced-intensity conditioning while factors predictive of worse outcomes were age >40 years old, unfavorable karyotype, active GVHD at relapse and previous HSCT from a mismatched unrelated donor or cord blood. In a study by the European Bone Marrow Transplantation society (EBMT) on 2815 patients transplanted with a reduced-intensity conditioning (RIC) regimen between 1999 and 2008, the incidence of relapse was 32%. Relapsed patients were treated with chemotherapy (mild or intensive) alone or followed by donor lymphocyte infusions (DLIs), DLIs alone or second HSCT. Two-year overall survival was 14% for the entire population. The factors associated with a better survival were absence of acute GVHD at relapse (*p* < 0.01), relapse more than 5 months after transplant (*p* < 0.001) and low tumor burden (i.e., an infiltration of bone marrow by blast cells inferior to the median of 27%). Two-year survival for patients having three and 0–1 risk factors were respectively 4% and 32%. Of interest, among patients reaching complete remission (CR) after “salvage” chemotherapy, the better outcome was associated with a cellular-based intervention (DLIs or second HSCT) as consolidation after CR. Two-year OS was 55% for patients receiving consolidation with cellular therapy versus 10% for those treated with chemotherapy alone [1]. Conventional treatments for relapse of AML after allogeneic HSCT, in addition to the suppression of immunosuppressive therapy, are chemotherapy (low-dose or intensive), DLIs and/or second transplantation. The choice of the most appropriate treatment has to be based on the patient’s and the disease’s characteristics such as performance status, comorbidities, the intensity of pretreatment, absence or presence of GVHD and chances of long-term survival based on the prognostic risk of the disease.

During the last two decades, the hypomethylating agents (HMAs) 5′-Azacytidine and decitabine entered the therapeutic armamentarium. HMAs, in addition to direct anti-leukemic activity, demonstrated a spectrum of immune-modulatory properties enhancing the graft-versus-leukemia (GVL) effect and justifying their use in the setting of the allogeneic hematopoietic transplant [5]. Moreover, a better understanding of the molecular mechanisms underlying leukemic relapse and the recent insights on the biology of the leukemic immune escape from the “GVL effect” expanded the clinical scenario to novel drugs towards molecular or immunological targets. Recent trials investigated the role of novel targeted drugs in the setting of relapsed-refractory AML and some of them have shown promising results in the maintenance after allogeneic HSCT [6,7]. In the present review, we discuss the current knowledge on the pathogenesis, prophylaxis and treatment of relapse of AML after HSCT, highlighting the main evidence regarding the clinical management of relapsed patients and reviewing the most relevant clinical studies published to date in support of such evidence.

## 2. Biological Bases of Leukemia Relapse Following Allogeneic Bone Marrow Transplantation

Treatment of acute leukemias relapsing after HSCT is an unmet clinical need. Relapse remains the major cause of HSCT failure [8]. At diagnosis, mutations in WT1, FLT3, DNMT3A and TP53 are associated with an increased risk of relapse post-transplant [9]. The mechanisms underlying post-HSCT relapse are mostly based on the selection of subclones resistant to the graft-versus-leukemia (GVL) effect. Enhanced exome sequencing was recently performed in patients with AML relapsed after HSCT. In this small sample—15 patients—the acquisition of previously unknown AML-specific mutation was uncommon, while down-regulation of major histocompatibility complex (MHC) class II genes was observed [10]. Therefore, mechanisms of relapse post-HSCT might be different from those of the post-chemotherapy relapse and are mostly based on the evasion from T-cell alloreactivity. Moreover, in vitro studies showed that the expression of HLA class-II molecules returned to normal levels when post-HSCT relapse AML cells were exposed to interferon-γ. This suggests that the down-regulation of HLA class-II molecules depends on epigenetic mechanisms and might explain the activity of hypomethylating agents in preventing and treating post-transplant disease relapse [11].

Mechanisms of immune evasion include the genomic loss of the mismatched HLA haplotype, the production of anti-inflammatory factors and/or the loss of pro-inflammatory cytokine production, the immunosuppression by immune-checkpoint ligand expression, the release of metabolically active enzymes and the acquisition of novel driver mutations [12].

The genomic loss of the mismatched HLA haplotype has been observed as an important mechanism of immune evasion in the HSCT from a haploidentical donor [13] and accounts for 33% of the relapse rate [14]. Rarely, it was also reported in the relapse following HSCT from a matched-unrelated donor (MUD) [15]. This seems to be due to a *de novo* mutation in residual leukemic cells after a long phase of immune equilibrium [16] 7w. The loss of the mismatched HLA haplotype occurs through a mechanism of copy-neutral loss of heterozygosity [12]. The incompatible HLA alleles are lost without decreasing the overall expression of HLA class I molecules. In this way, both T-cell and NK cell-mediated responses are inhibited, since T-cells usually activate following the recognition of the mismatched HLA haplotype and NK cells activate in the absence of self-HLA class I on leukemic cells. 

The documentation of the mismatched HLA haplotype loss at relapse is clinically important. Indeed, in this particular case, early withdrawal of the immunosuppressive treatment and the administration of donor lymphocyte infusion (DLI) in order to enhance the GVL effect might be ineffective and potentially harmful. Moreover, the loss of the HLA haplotype at relapse should be considered in the case of a second HSCT, since re-transplantation from the original donor would be useless, while the use of alternative donors is suggested. 

Studies in the non-transplant setting suggest that another possible mechanism of immune evasion is the modulation of the production of anti-inflammatory and pro-inflammatory cytokines by leukemic cells [17]. 

For example, IL-15 is a pro-inflammatory cytokine that is produced by myeloid progenitors in normal conditions [18]. A decreased production of IL-15 might reduce the T-cell and NK cell activation, thus reducing the GVL effect. Indeed, low levels of IL-15 measured at day +14 after HSCT were associated with malignancy relapse [19]. Furthermore, in vitro studies on FLT-3 mutated AML cells showed a decreased production of IL-15; studies in animals showed that the FLT-3 inhibitor sorafenib increases IL-15 production by FLT-3 AML cells through the inhibition of the transcriptional factor ATF4 causing activation of the IRF7–IL-15 axis in leukemia cells [20]. A phase I single-arm multicenter study reported increased activation of T-cells and NK cells in patients who relapsed post-HSCT after administration of ALT-803, an IL-15 super-agonist complex [21]. 

The role of inhibitory immune checkpoint in the relapse post-HSCT is the subject of research. The down-regulation of immune-regulatory molecules including PDL-1 on AML cells at the time of relapse post-HSCT was observed [22]. PD-1 inhibitors could have an important role in the treatment of post-HSCT acute leukemia relapse. Nivolumab has been used to treat post-HSCT relapse of Hodgkin lymphoma. In this setting, it is shown to be an effective therapeutical option but also to increase the risk of GVHD, suggesting that it acts by enhancing the GVL effect [23]. In the non-transplant setting, nivolumab was used in combination with azacytidine in a phase II clinical trial in patients with relapsed/refractory AML [24].

Other possible mechanisms of immune evasion described in the non-transplant setting include the production of IDO-1, arginase, CD39 and CD73 by leukemia cells, leading to an increased level of kynurenine and adenosine that have immunosuppressive properties and to the depletion of arginine which is essential to T-cells [25,26,27,28]. 

Understanding the biology underlying the recurrence of acute leukemia after allogeneic bone marrow transplantation is of primary importance in order to introduce new strategies for its prevention and treatment. At relapse, reassessment of the mutational profile of the disease on bone marrow or peripheral blood samples should be performed in order to identify molecular alterations potentially targeted by new agents. Moreover, although outside of clinical practice for most centers, the study of immune-escape mechanisms might be of help in tailoring therapeutic interventions to the patient. 

## 3. The Role of MRD Monitoring

Measurable (“minimal”) residual disease (MRD) is defined as the post-therapy persistence of leukemic cells at levels below the morphologic detection limit [29]. 

Mounting evidence indicates that the presence of MRD is a strong, independent prognostic marker of increased risk of relapse and of shorter survival in patients with acute leukemia compared with patients with a negative MRD test [30].

MRD assessment primarily involves the determination of leukemia-associated immunophenotypic patterns (LAIP) using multiparameter flow cytometry (MCF) and the polymerase chain reaction (PCR)-based evaluation of expression levels of leukemia-related genes (specific reciprocal gene rearrangements and other mutation types). In addition, next-generation sequencing and digital PCR may further enrich current MRD-detection methods [31].

Adding the MRD evaluation to other post-treatment assessments could be of help in guiding the post-remission treatment strategies by identifying patients at high risk of relapse who might benefit from pre-emptive therapy. Several studies have clearly shown that treatment is more effective if at molecular relapse with a low disease burden than at overt relapse [32].

### 3.1. Multiparameter Flow Cytometry (MFC)

MFC uses panels of fluorochrome-labeled monoclonal antibodies to identify aberrantly expressed antigens located on leukemic cells. By using combinations of multiple monoclonal antibodies, the sensitivity of MFC is increased to detect 10–3 to 10–5 leukemic cells within the white blood cell compartment [33].

MFC assessment after transplantation (day +100) discriminated different risk populations in AML patients. Overall survival (OS) was 73% vs. 25% after 4 years among patients with low (<10–3) vs. high (≥10–3) MRD at day +100 after transplantation (*p* = 0002) [34].

MFC for well-defined leukemia-associated immunophenotype patterns (LAIP) has broad applicability (<90%) and high specificity. However, the leukemic phenotype is not necessarily stable over time (e.g., the initial LAIP may be lost in the course of the disease), and there remains limitations of this method due to a lack of comparability and reproducibility among different laboratories, the use of different instruments, fluorophores and operating procedures that require further standardization [35]. Current guidelines recommend to use, in the follow-up of the patient the combined approach of detecting the LAIP and the different from normal (DfN) phenotypes (which individuate aberrant differentiation or maturation phenotype) [36].

### 3.2. Real-Time Quantitative PCR (RT-qPCR)

Molecular assessment of MRD can be performed by monitoring mutated genes [29], fusion-gene transcripts [30] and overexpressed genes [31]. The PCR-based techniques applied to the quantitative measurement of these markers currently represent the standard of care with the highest sensitivity (down to 1:10^6^ cells) and specificity [36].

About 30% of patients with normal-karyotype AML have an nucleophosmin-1 (NPM1) gene mutation. Several groups have already demonstrated the predictive value for relapse of a persistent NPM1-mutation in peripheral blood (PB) and bone marrow (BM) in patients in complete remission after conventional chemotherapy [37]. NPM-1 mutation is recommended in clinical practice as MRD-marker [36].

Molecular testing for FLT3 mutations is part of the diagnostic setting at the first diagnosis and offers essential prognostic information. However, it is not routinely used to monitor MRD after HSCT due to its relative instability during the course of the disease [38].

Other mutations (DNMT3A, IDH1/2, TET2, ASXL1), because of their relatively low frequency, have not been studied in larger cohorts and lack broad applicability [38].

In about 20% of patients with AML, distinct fusion genes are approachable for MRD monitoring, with most of them expressed by the core-binding factor (CBF) AML (RUNX1-RUNX1T1 and CBFB-MYH11 fusion genes). Several reports have already demonstrated that MRD positivity detected by quantitative PCR is predictive for imminent relapse in patients with CBF leukemias after conventional therapy [39].

Overexpression of Wilms tumor 1 (WT1) mRNA is present in about 90% of patients with AML and 50% of patients with MDS. Thus, monitoring of WT1 is broadly applicable in a large proportion of AML and MDS patients [40]. Furthermore, WT1 expression is measurable in peripheral blood with even higher sensitivity and specificity than in bone marrow, thereby facilitating the patient’s comfort in contrast to other molecular methods which require a myeloaspirate to gain a comparable sensitivity. However, WT1 lacks specificity and is not routinely recommended for MRD monitoring unless there is no alternative marker, including flow-cytometric markers [36]. When WT1 expression is used for MRD monitoring, a validated ELN assay offers a reproducible cut-off and comparable results among different laboratories [41]. 

The advantages of real-time quantitative polymerase chain reaction (RT-qPCR) are the broad applicability and the high sensitivity (superior to MFC); it is well standardized and maybe run by a certified laboratory with expertise in RT-qPCR. 

However, given the limited available molecular targets, RT-qPCR assessment of MRD is applicable to only 50% of AML cases and less than 35% in older patients (whereas MFC can detect MRD in 90% of patients when a comprehensive antibody panel is used) [42]. Limitations of the RT-qPCR-based assays are their dependence on specific mutations, requiring individual reference standard curves based on target serial dilutions; in addition, the results of RT-qPCR may take multiple days. It is an expensive assay and requires a high level of expertise [43].

### 3.3. Digital PCR

Digital droplet PCR (ddPCR) is a biotechnological refinement of the conventional qPCR methods that can quantify and clonally amplify the nucleic acid strands directly. Compared to qPCR, it is more precise, reproducible and accurate. For example, digital PCR can detect a variety of NPM1-mutation subtypes without the need for multiple plasmid standards [44]. A disadvantage of ddPCR is that for each single mutation a specific assay has to be developed [36]. 

### 3.4. Next-Generation DNA Sequencing (NGS)

Next-generation DNA sequencing technologies, which allow parallel and repeated sequencing of millions of small DNA fragments, can be used to evaluate a few genes or an entire genome [45]. The ability of NGS to assay large numbers of mutated genes could be of help in tracing the evolution of malignant clones. Several studies have demonstrated the feasibility of NGS to monitor the mutations for which targeted therapies are available, such as FLT3-ITD [46], IDH1/2 [47] and the mutations having prognostic relevance, such as CEBPA and NPM1 in patients with AML [48]. The NGS may provide further information for relapse, particularly when MRD evaluated by conventional methods is negative. Recently, Heuser and co-workers showed that MRD measured by NGS-based techniques at days +90 and +180, together with MRD measured pre-HSCT, is highly predictive for relapse and of overall survival [49]. However, this technique is time-consuming and costly and is not recommended for MRD measurement outside of clinical trials [36,45]. 

### 3.5. Chimerism Analyses

The analysis of donor/recipient chimerism is the standard practice to monitor donor cell engraftment and can be performed in all patients after HSCT. Chimerism analysis detects the host-derived hematopoiesis on the basis of the genomic differences of highly variable loci between the recipient and the donor. A mixed donor/recipient chimerism does not equate directly to the relapse of the leukemic clone in all cases. However, in malignant disorders such as AML, a decrease of donor chimerism is often associated with disease recurrence [50]. In the absence of other markers, chimerism analysis has been suggested as a surrogate of MRD monitoring. The variant allele-specific qPCR method for detection of small insertion and deletions is more sensible than the short tandem repeats (STR) analysis and it should be preferred for MRD monitoring [51].

The therapeutical options in the case of MRD positivity detection following HSCT will be discussed in Section 6 (pre-emptive therapy).

## 4. Treatment of AML Relapsed after HSCT

### 4.1. Donor Leukocyte Infusions (DLIs)

The use of DLIs, over 30 years ago, was described in the treatment of chronic myeloid leukemia (CML) relapsing after HSCT [52]. Since then, several studies have reported on DLIs in other clinical settings. 

Concerning the mechanism of action of DLIs, studies conducted in CML relapsing after HSCT have shown that in patients responsive to DLIs, lymphocytes T-CD8+ resident in the microenvironment of the bone marrow receive a proliferative stimulus after DLIs which triggers a cell-mediated response against neoplastic hematopoiesis [53]. Gene-expression profile (GEP) analysis has demonstrated that genetic pathways of T-cell exhaustion in T-CD8+ cells resident in BM of relapsing patients are down-regulated in response to DLI. These data are suggestive that novel donor lymphocytes infused might provide signals of induction and activation to exhausted T-lymphocytes pre-existing to DLI [54]. 

In acute myeloid leukemia, the role of T-cell exhaustion has been demonstrated in the pathogenesis of the relapse after HSCT. The T-lymphocytes expressing high levels of the inhibitory molecules PD-1 and TIM-3 are functionally deficient in secreting cytokines such as Il-2 and TNF-alpha and have been associated with relapse [55]. In a prospective study of AML patients relapsing after the HSCT, the authors confirmed that T-CD4+ and T-CD8+ cells of the relapsing patients express high PD-1 and TIM3 levels and that the T-exhaustion was correlated with a marked inhibition of the proliferative capacity and of the cytokine production. Such functional impairment involved, particularly, the subsets of antigen-experienced cells (T-effector memory and T-central memory) [56]. Relapse was treated with administration of salvage chemotherapy followed by DLI (that was G-CSF-primed and associated with immunosuppressive prophylaxis of GVHD). In the patients responsive to treatment, the T-cell exhaustion markers were down-regulated and the functional defects of T-CD4+ and T-CD8+ lymphocytes were reversed, restoring their proliferative capacity and cytokine production [56]. 

From the analysis of the TCR-diversity of the CD8+ lymphocytes in patients receiving DLI, it emerged that in patients developing a GVL effect, a minor TCR-diversity is associated with a lower reactivity versus minor histocompatibility antigens (mHAs) of the non-hematopoietic tissues and a lower GVHD [57]. 

More recently, a German study analyzed the variation of the TCR-αβ repertoire following the administration of DLIs in a cohort of relapsing patients after HSCT (most of them affected with AML). They have found that in patients responding to DLIs, the absolute number and the frequency of T-CD8+ cells remained constant while their TCR-diversity was reduced (but not of the CD4+ or the entire CD3+ TCR-repertoire), thus suggesting a clonal expansion of some specific T-CD8+ clones. Absence of such expansion in patients not achieving a GVL effect after DLIs was predictive of relapse in a median of 11 months before diagnosis. The clonal expansion correlated to the GVL effect involved clones both pre-existing to DLI and newly introduced with the DLI [58]. 

Concerning the treatment schedule, a dose-escalation protocol is preferred to a single dose in order to mitigate the risk of GVHD without compromising GVL [59]. 

There is no consensus among authors on the most appropriate starting dose and the escalation protocol. The choice depends on clinical and biological variables such as the donor type, the time elapsed from HSCT and the clinical context (i.e., treatment of relapse or as prophylaxis or pre-emptive therapy). 

DLIs administered within three months after HSCT are at high risk of GVHD and should not be recommended except in the case of clinical relapse. Within six months from HSCT, a progressive replacement of antigen-presenting cells (APCs) compartment from host to donor origin occurs [60]. This might be the reason why the risk of GVHD associated with administering a higher dose of CD3+ lymphocytes away from the transplant is lower than when they are administered shortly (within six months) after the transplant [61]. When used as a treatment of overt relapse, the first dose of CD3+ cells infused of 1 × 10^6^/Kg (recipient’s body weight) and 1 × 10^7^/Kg followed by an escalation to 5 × 10^6^–10^7^/Kg and 1 × 10^7^–10^8^/Kg every 4–6 weeks, respectively, from a matched-unrelated and from a matched-related donor might be considered [61].

A lower first dose seems appropriate when DLIs are from haploidentical or related-mismatched donors. In a cohort of 40 patients (16 affected with AML) who underwent haploidentical transplant, Zeidan proposed a first dose of 1 × 10^5^/Kg CD3+ followed by an escalation to 1, 5 and 10 × 10^6^/Kg, respectively. However, no clinical response nor significant GVHD were observed after 1 × 10^5^/Kg CD3+ and the majority of patients received 1 × 10^6^/Kg CD3+ as first DLI with an incidence of acute GVHD of 25% as that expected from DLIs from matched donors [62]. 

The use of therapeutic DLIs from haplo donors should be preceded by an evaluation of the potential HLA-loss, in which case the therapeutic DLI is inappropriate [63].

Recently a consensus expert panel on haploidentical DLIs recommended a first therapeutical dose of CD3+ 1 × 10^6^/Kg, preceded by cytoreductive therapy and followed by an escalating protocol of 0.5–1 log_10_. Instead, as pre-emptive DLI from haplo-donors, the same panel recommended a dose of CD3+ 1 × 10^5^/Kg [63]. 

Retrospective analysis and registry data indicate that DLIs, when used alone, provide greater chances of treatment success when the leukemic burden is low, or the relapse is at the molecular level [64] and the biological rationale supporting this clinical observation can be guessed from the immunological mechanism of action of DLIs. The same retrospective analysis suggests that DLIs should be used to consolidate the results achieved by conventional chemotherapy or epigenetic therapy, which, instead, if used alone, might have a low rate of success. 

In a large retrospective analysis from the EBMT, Schmid and co-workers compared 228 patients who received DLIs as part of the salvage treatment for their first relapse with 171 patients in which DLIs were not included in the treatment protocol. 

Two-year survival of patients was 21% and 9% respectively for DLI and no-DLI patients (*p* < 0.0001). In a multivariate analysis, factors associated with overall survival (OS) were aged < 37 years old (*p* < 0.008), relapse later than five months after HSCT (*p* < 0.0001) and having received DLIs (*p* = 0.04). 

Among the DLI-patients factors associated with a better OS were low tumor burden at relapse defined as a BM blast infiltration < 35% (*p* = 0.006), female sex (*p* = 0.02), favorable cytogenetic risk profile (*p* = 0.004) and complete remission (CR) status prior to DLIs (*p* < 0.0001). 

Two-year OS was 56% and 15%, respectively, for patients receiving DLIs in CR and not in CR (*p* = 0.0005) [64]. 

A retrospective registry study from the Japan Society for HSCT analyzed 143 patients treated with DLIs between 1991 and 2011. Two-year OS for the whole cohort of patients was 17%. In a multivariate analysis, factors associated with a better outcome were relapse later than five months after HSCT (*p* = 0.02) and CR at the time of DLIs (*p* = 0.002). The 2-year OS for patients relapsed more than five months after HSCT was 100% [65].

Subsequently, Miyamoto published a retrospective analysis on 414 patients affected with various hematologic neoplasms (184 AML patients) and receiving DLIs [66]. 

Eighty-four percent and sixteen percent of patients, respectively, had received DLIs as treatment of overt relapse or cytogenetic/molecular relapse. The variables correlated with best response to DLIs were occurrence of GVHD (*p* = 0.024), DLIs used as pre-emptive treatment instead of for overt relapse (*p* = 0.08) and diagnosis of CML (*p* = 0.007). CR for patients treated pre-emptively and for overt relapse was, respectively, 57% and 20% (*p* < 0.001) [65].

More recently, the Gruppo Italiano Trapianto di Midollo Osseo (GITMO) has published the results of a survey on the use of DLIs among 34 centers in Italy between 2010 and 2015 [67]. 

The survey included 152 acute leukemia (AL) patients [180 (70%) AML, 68 (30%) ALL, 4 (2%) biphenotypic acute lukemia] and the median age was 45 years old. Donor type was distributed as follows: HLA-identical 98 (39%), unrelated 102 (40%), 1 antigen-mismatched-related 3 (1%) and haploidentical 49 (20%). Indications to DLIs were treatment of relapse in 73%, mixed donor-recipient chimerism in 17% and pre-emptive/prophylaxis of relapse in 10% of patients. The proportion of the patients receiving treatment (conventional chemotherapy, targeted therapy or radiotherapy) prior to DLIs was 60%. 

Incidence of grade I–IV acute GVHD (aGVHD) was 30% (grade III–IV aGVHD: 3%) and of chronic GVHD (cGVHD), 47% (severe cGVHD 12%). The rate of aGVHD distributed according to the different types of the donor was 9% from HLA-identical siblings, 21% from unrelated and 28% from the haploidentical donor. Eleven percent of patients experienced grade III–IV hematological toxicity. The DLI-related mortality was 9%. 

One-year, three-year and five-year OS were, respectively 55%, 39% and 35%. In a multivariate analysis, factors associated with a better OS were pre-emptive/prophylactic use of DLIs instead of therapeutic DLIs (3-year OS respectively, 55% vs. 32%; *p* = 0.000) and having received multiple infusions than a single infusion (*p* = 0.05). Over two years, the longer time between HSCT and the first DLI was associated with a better OS (*p* = 0.003). Of note, the rate of CR and stable disease after DLIs from haploidentical donors was inferior to that with DLIs from unrelated donors (33% vs. 78%; *p* = 0.03). The response rates to DLIs from HLA-matched-related and haploidentical donors were not different [67]. 

Researchers from the University People’s Hospital of Bejing performed a retrospective comparison between 50 patients receiving chemotherapy combined with DLIs and 32 patients receiving chemotherapy alone as treatment of AL relapsed after haploidentical T-replete HSCT. The diagnosis was AML in 45 and ALL in 37 patients, respectively. DLIs were “modified-DLIs”, that is, G-CSF-mobilized DLIs followed by immunosuppressive therapy with Cyclosporine A (CyA) and Methotrexate to prevent GVHD (as discussed below). The DLI was administered as a “single dose”. The median dose was 4.4 × 10^7^/Kg CD3+. 

CR was significantly higher for patients receiving chemotherapy followed by DLI than for those receiving chemotherapy alone (64% vs. 12% respectively; *p* = 0.000), although patients treated with the combination had a higher percentage of blasts infiltrating BM at relapse (in median 50% vs. 34%, respectively; *p* = 0.001). 

One-year post-intervention OS and DFS were both, respectively, 36% for the chemo + DLI group and 0% for the chemotherapy alone group (*p* = 0.000). 

In a multivariate analysis, the incidence of cGVHD (*p* = 0.000) and chemotherapy prior to DLIs instead of chemotherapy alone (*p* = 0.037) were associated with a lower relapse rate [68]. 

In summary, taken together, the data by the retrospective studies about DLIs indicate that the clinical efficacy of this therapeutic intervention might be better when it is administered in complete remission or when it is used for the treatment of a minimal disease or a low leukemic burden. In patients with overt relapse, the results of DLIs might be improved by administering prior adjuvant therapy in order to reduce the leukemic burden. Hematological malignancies exhibiting slow-growing features, as for late-relapsing diseases after HSCT, may provide more chance to be sensitive to the GVL effect triggered by the DLIs. 

### 4.2. Hypomethylating Agents in Treatment of AML Relapse: Mechanism of Action

In the last two decades, the introduction of hypomethylating agents (HMAs), decitabine (DAC) and its pro-drug 5′-azacitidine (5′-AZA) in the clinical practice have modified the landscape of the therapeutic options for the treatment of myeloid malignancies. These drugs, by demethylating the DNA and inducing the transcription of silenced genes in the nucleus, together with the histone-deacetylase inhibitors (HDACi), are considered epigenetic modifiers. 

HMAs, besides their direct anti-leukemic efficacy, have shown a spectrum of activities on the leukemic microenvironment and immunological reaction by the anti-leukemic T-cells and NK cells. These properties rendered HMAs a class of drugs desirable in the setting of post-transplant relapsed disease. 

It has been shown that HMAs, by demethylating the promoter region of silenced genes, may enhance the expression of tumor-specific antigens such as cancer-testis antigen [69]. Moreover, hypermethylation has been involved in the HLA-loss mechanisms underlying the immune escape of tumor cells, and HMAs may induce re-expression of HLA class I molecules [70]. In ovarian cancer, it has been shown that inhibition of DNA-methyl transferase by azacytidine induces a type-I interferon response and apoptosis of cancer cells by up-regulation of hypermethylated endogenous retrovirus that triggers an antiviral response [71]. In NK cells, DNA demethylation is responsible for the transcription of genes encoding for the killer Ig-like receptors (KIR) by enhancing their antitumoral activity [72]. 

Concerning the immune-modulatory activity of HMAs, azacytidine has been shown, in mice, to induce foxp3 expression on T-CD4+ cells by converting them in T-CD4+/CD25+/Foxp3+ cells (T-regulatory—T-regs), capable of mitigating GVHD while preserving GVL [73]. In a murine model of leukemia and MHC-mismatched allogeneic transplantation, 5′-AZA and T-CD4+/CD8+ conventional lymphocytes (T-Conv) when administered concurrently were more effective in reducing the tumor burden than when administered apart [73].

The immunomodulatory activity of 5′-AZA has also been demonstrated in humans. In a cohort of 27 high-risk patients treated with 5′-AZA and compared with a control group, Goodyear and co-workers demonstrated an expansion >3 log_10_ than controls of the T-CD4+/CD25+/Foxp3+ cells and of the tumor-antigen-specific T-CD8+ cells after completion of more than three cycles of treatment. The phenotype of the tumor-specific T-CD8+ cells was that of the “effector-memory” T-cells (CD45RA-, CCR7-). Functional analysis also revealed that in response to 5′-AZA, tumor-specific T-CD8+ cells improved their secretory activity of IL-2, IFN-g and TNF-a [74]. 

### 4.3. HMAs in the Treatment of Overt Relapse: Clinical Results from Retrospective Trials

Since the introduction of HMAs in clinical practice, several case series or retrospective studies have been published on the use of such agents in treating the relapse of myeloid malignancies after HSCT. The retrospective studies report an overall response rate ranging from 19% to 54% (CR rate ranging from 7% to 27%). The reported 2-year OS from starting the treatment ranges from 11% to 27% [75,76,77,78,79,80,81,82]. However, the data from the retrospective studies are difficult to interpret because of their extreme heterogeneity in the patient’s characteristics, schedule of treatment, response criteria and period of treatment. Moreover, most of them include both AML and MDS patients. 

Craddock and co-workers, extrapolating data from the EBMT database on 181 patients (116 AML, 65 MDS) treated after HSCT for hematological relapse, developed a prognostic score predictive of response 5′-AZA. The risk factors associated with low response rate were relapse earlier than 6 months from HSCT and the percentage of blast cells in the BM ≥20% at the time of relapse. The probability of CR after 5′-AZA ranged from 8% for patients with two risk factors to 34% for patients without risk factors (*p* = 0.0019). Two-year OS ranged from 3% for score 2 to 37% for score 0 (*p* < 0.00001). The median 2-year OS of patients achieving CR was 48% with respect to 12% in the whole cohort. This analysis excluded patients receiving 5′-AZA for pre-emptive therapy or prophylaxis [83]. 

Therapy with HMAs for overt relapse of AML or MDS after HSCT was retrospectively compared with the conventional cytotoxic chemotherapy in a cohort of 100 patients. Seventy-three patients received purine analog- or anthracycline-containing regimens while twenty-seven patients received HMAs (5′-AZA or DAC). The rate of CR/CRi was 51% and 19% respectively for chemotherapy and HMAs (*p* = 0.004), and median PFS was 4.9 months vs. 3.8 months respectively for chemotherapy and HMAs (*p* = 0.02). Of note, 56% of patients in the chemotherapy group and 33% in the HMAs group also received consolidation with DLIs, and in both groups, the addition of DLIs resulted in improved response rate and PFS [79]. 

Taken together the retrospective analysis indicate that selected patients may benefit more than others from HMAs in the treatment of relapse post-HSCT. The results are better when the diagnosis is MDS rather than AML, when relapse is at the molecular level rather than overt relapse, when tumor burden is low rather than high and when relapse occurs later than six months from HSCT. To date, there is no clear advantage of HMAs over cytotoxic chemotherapy in controlling the disease. The response may be better if it is consolidated by a cellular-based intervention. 

### 4.4. HMAs in the Treatment of Overt Relapse: Clinical Results from Prospective Trials 

HMAs have been the subject of several prospective studies investigating their role, alone or in combination with DLIs, in the treatment of overt relapse or in the pre-emptive/prophylactic setting. 

Schroeder and co-workers designed a phase II study to evaluate the response to 5′-AZA as treatment of relapse of AML or MDS after HSCT named AZARELA trial. The study enrolled 30 patients (28 AML and 2 MDS) assigned to receive up to 8 cycles of 5′-AZA 100 mg/m^2^ for five days in a 28-days cycle followed by escalating-dose DLIs every second azacytidine cycle. A median of 3 cycles was administered, and 22 patients received at least one DLI. Eight of the thirty patients completed the scheduled treatment. Overall response was 30% (9/30) with 23% CR (7/30) and 7% (2/30) PR. 

In the responding patients, the median duration of response was 777 days. In the whole cohort, the median OS was 117 days (not reached for patients achieving CR and 83 days for patients not achieving CR - *p* < 0.001) [84]. 

Researchers from the University of Missouri designed a phase I “dose-finding” study evaluating the immune-modulatory properties of 5′-AZA administered after the DLIs to mitigate the GVHD. Eight patients entered the study, and two dose levels of 5′-AZA were evaluated: 45 mg/m^2^ and 75 mg/m^2^ on days +4, +6, +8 and +10 post-DLI. No dose-limiting toxicity (DLT) occurred. The response was reached in 6/8 patients (4 CR/CRi and two cytogenetic remissions). Median DFS and OS were 2.9 and 12.5 months [85]. 

The combination of 5′-AZA with lenalidomide (LEN) was investigated in an open-label, dose-finding, phase I study (VIOLA trial). Twenty-nine patients, affected with AML (24) or MDS (5) and relapsing after HSCT entered the study. The patients received 5′-AZA alone or combined with LEN. 5′-AZA was administered at a 75 mg/m^2^ dose for seven days, while LEN was subdivided into five dose levels (from 2.5 mg/day to 25 mg/day on days 10–30 of a 42-day cycle). The dose of 25 mg/day was determined as the maximum-tolerated dose (MTD) of LEN in association with 5′-AZA. The median number of administered cycles per patient was three. Seven of the fifteen patients who received at least three cycles responded (CR/CRi/PR). Median OS was ten months in not-responders and 27 months in responders [86]. 

In order to identify risk factors predictive of response to 5′-AZA in AML and MDS patients affected with early relapse post-HSCT, authors at the Fred Hutchinson Research Center designed a prospective study enrolling patients who relapsed within 100 days from HSCT. Thirty-nine patients entered the trial and received 5′-AZA at 75 mg/m^2^ for seven days in a twenty-eight-day cycle for at least six cycles. The response was achieved after a median of six cycles in 30% of patients (12/30: 3 CR, 9 PR). In a multivariate analysis, pre-HSCT induction treatment with Cladribine, cytarabine and Mitoxantrone + G-CSF or with FLAG-IDA (fludarabine, cytarabine, idarubicine, G-CSF) were associated with a better probability of response and survival (*p* = 0.004). The 2-year OS was 25% in the whole cohort and 70% in patients achieving CR or PR. The author’s argument is that the previous intensive chemotherapy, by exerting a “de-bulking” effect and altering the clonal composition of the disease, might have rendered it more sensitive to 5′-AZA [87]. 

Recent studies demonstrate a clinical activity of the BCL-2 inhibitor venetoclax (VEN) in combination with HMAs in elderly patients and in relapsed/refractory AML [88,89]. A very recent study was designed to treat AML patients who relapsed after HSCT with the combination of 5′-AZA and VEN followed by DLIs. In this study, the authors utilized 5′-AZA at a 75 mg/m^2^ for five days in a 28-day cycle up to six to eight cycles. VEN started at a dose of 100 mg/day in the first week and was increased by 100 mg every week to a maximum of 400 mg/day that was the maintenance dose. 

A single “G-CSF-mobilized” DLI was administered on day +6. No immunosuppressive therapy was given after DLI. If GVHD did not develop, DLI was repeated after three months. The response was achieved in 16/26 patients (61%) and were CR in 7 and PR in 9 patients. The median number of cycles to achieve the remission was two (1–2). Median EFS and OS were, respectively, 120 days and 284 days [90]. 

The NCT02472691 trial is an ongoing prospective, open-label, single-arm, phase II trial evaluating the 5′-AZA in combination with DLI and LEN as treatment of relapse of MDS and AML with MDS-related changes after HSCT. 

5′-AZA will be administered at a 75 mg/m^2^/day dose for seven days in a twenty-eight-day cycle up to eight cycles. DLIs will be administered after the fourth, sixth and eighth cycle if no GVHD occurs. The study includes a part of dose-finding in which LEN is administered at 2.5 mg/day to 10 patients and a second part in which LEN is escalated to 5 mg/day on days 1–21 of each cycle if DLT does not occur. The treatment is stopped if grade II–IV aGVHD occurs.

The primary outcomes are the number of participants experiencing adverse events (AEs) and the severity of AEs. Secondary end-points include response rate, OS, duration of response and incidence of GVHD. 

Data from the prospective studies on HMAs in the post-HSCT indicate that the treatment of overt relapse with such agents is feasible. The response should be expected in about one-third of the patients. In the responding patients, the response should be expected after three to four cycles of therapy when HMAs are used alone. The combination of HMAs with other synergizing agents appears promising but data from combination studies are still limited and need further investigation. Given the immune-modulatory properties of HMAs, their potential synergy with DLIs should always be exploited when clinically feasible. 

With respect to conventional intensive “salvage” regimens, the non-hematological toxicity profile of HMAs is better, which might be advantageous over chemotherapy in the prevision of a cellular-based therapy (i.e., DLIs or second HSCT). Given their activity on DNA derangement of suppressed genes and their immune-modulatory effect, the role of the epigenetic modifiers seems more attractive when used as maintenance or pre-emptive treatment after HSCT. 

The results of the clinical trials evaluating the role of HMAs in the maintenance and pre-emptive therapy after HSCT will be discussed later. 

Table 1 summarizes the results of selected studies on the use of HMAs in the treatment of relapse post-HSCT.

### 4.5. Second Allogeneic Hematopoietic Transplantation 

The high risk of morbidity and of mortality associated with a second allogeneic HSCT and the particular frailty of the patients who relapse after a first transplant make such intervention applicable only in very selected cases. However, the progress made in the last two decades in supportive care, in designing more effective RIC regimens, in the prophylaxis of GVHD and in the utilization of haploidentical donors has made it possible to offer a second transplant to an ever-increasing number of patients. 

The data on the second HSCT derive from the retrospective analysis. 

A registry study from the EBMT database analyzed the outcome of 179 patients affected with AL (AML: 132, ALL: 46, unclassifiable leukemia: 1) that underwent the second HSCT between 1998 and 2009 for relapse after a previous HSCT. The median age at second HSCT was 39 years old. 

The authors studied the role of the change of donor. Among the 75 recipients of the first HSCT from a matched-related donor (MRD), the donors for the second HSCT were, respectively: the same MRD in 38 (51%), a different MRD in 8 (11%) and an unrelated donor (UD) in 29 (38%). Among the 104 recipients of the first HSCT from an unrelated donor, the donors for the second HSCT were respectively: the same UD in 44 (42%) and a different UD in 60 (58%). The median 2-year OS and LFS from the second HSCT were, respectively, 25% and 21%. After the second HSCT, the cumulative incidence of grade II–IV aGVHD was 53%. The median incidences of limited and extensive cGVHD were 23% and 29%, respectively. 

Factors predictive of a better OS from second HSCT were: previous CR longer than six months after first HSCT (*p* < 0.001), CR at second HSCT (*p* = 0.006) and first HSCT from an MRD than from a UD (*p* = 0.042). There was a clear advantage from changing donors only in the small subgroup of patients (*n* = 8) relapsing after a matched-sibling donor transplant and receiving the second HSCT from a different MRD (2-year OS 88%). In the subgroup of patients who received the first HSCT from a UD, the survival was superior if the second HSCT was from a different UD than from the same UD (2-year OS 20% vs. 11%; *p* = 0.037). However, the advantage was limited to patients that did not experience aGVHD or cGVHD after the first HSCT (*p* = 0.01) [91].

Similar results were reported by the Group Espa̴nol de Trasplante Hematopoyetico (GETH) in a retrospective study on 116 patients (median age: 38) who received second HSCT for relapse of myeloid malignancy (AML: 88) between 1979 and 2011. The second HSCT were from an MRD in 83%, from a UD in 11%, from a mismatched-related donor in 4% and from a syngeneic in 2% of the patients. The majority (70%) of patients had active disease at second HSCT. Five-year OS was 32%. 

Factors predictive of a shorter survival were active disease at second HSCT (*p* < 0.001), the time between the two transplants inferior to 430 days (*p* < 0.001), second HSCT from a donor different from an HLA-matched related (*p* = 0.03). 

Concerning the NRM after the second HSCT, the following factors were identified as bearing a poor prognosis: myeloablative conditioning (*p* = 0.01), active disease (*p* = 0.02), a second donor other than an HLA-identical sibling (*p* 0.009). The study failed to find a clear advantage in changing donor. However, using a second HSCT from an HLA-identical sibling instead from an unrelated donor was associated with better survival because of a lower NRM [92]. 

Duncan and co-workers, by analyzing data from the registry of the Center for International Blood and Marrow Transplant Research (CIBMTR) on patients surviving beyond one year after second HSCT, reported a 10-year OS, respectively of 55% and of 39% for children and adults. Incidence of cGVHD was, respectively 43% and 75%. Besides GVHD, more than half of the patients (63% of children and 55% of adults) also experienced other late side-effects such as growth disturbance and cataracts for children and cataracts and avascular necrosis for adults. Relapse, also among long-term survivors, continued to be a leading cause of mortality (10-year CIR, respectively: 34% and 32% for children and adults) [93].

A retrospective study from the MDACC on 91 patients receiving a second HSCT for AML relapse reported a 2-year OS, progression-free survival (PFS) and NRM, respectively, of 36%, 27% and 18%. The median age was 44 years old. Factors associated with inferior outcome after second HSCT were comorbidity index (HCT-CI) ≥2 (*p* = 0.01) and previous chronic GVHD (*p* = 0.001) [94].

In the last two decades, the use of haploidentical transplant has become more extensive, especially after the introduction of the “T-replete”-platform using cyclophosphamide 50 mg/Kg/day on days +3 and +4 (post-transplant cyclophosphamide—PTCy) [95]. 

More recently, the EBMT published the results of a retrospective comparison between the second HSCT from a matched-unrelated donor or haploidentical donor performed for AML relapse after a first HSCT. Five hundred fifty-six AML patients who received the second HSCT between 2006 and 2016 because of relapse after previous HSCT were analyzed. The median age was 46 years old. The cohort was divided into three groups based on the switch of donors:

(1) Switch from a first MRD or UD to a second HSCT from the same donor (same donor group)
(2) Switch from a first matched-related or unrelated donor to a second different matched-related or unrelated donor (second different matched donor)
(3) Switch from a first matched-related or unrelated donor to a second haploidentical donor (second haplo-group).

All the haploidentical transplants were T-replete with PTCy (70%), ATG (25%) or both (5%). Half of the patients (55%) had active leukemia at the second HSCT. Per inclusion criteria, all the second HSCT were performed within 300 days from relapse. 

Outcome did not differ among the three groups. Two-year LFS was 23.5, 23.7 and 21.6% respectively (*p* = 0.3) and 2-year OS was 36.4%, 28.7% and 23.3.%, respectively (*p* = 0.21). 

Predictors of shorter LFS were relapse within 6 months after first HSCT (*p* = 0.01), advanced age (*p* = 0.07), second haploidentical donor instead of the other donor-types (*p* = 0.07) and active disease at second HSCT (*p* = 0.002). 

Two-year NRM was, respectively 25%, 27% and 34% (*p* = 0.28), and there was a trend for a higher incidence of death due to infections in the haplo group (*p* = 0.08). 

Two-year relapse rate was 51%, 49% and 44%, respectively (*p* = 0.9). Factors associated with a lower relapse rate after second HSCT were relapse later than 6 months from previous HSCT (*p* = 0.03), first HSCT from an unrelated donor (*p* = 0.005), previous cGVHD (*p* = 0.05) and CR at second HSCT (*p* = 0.002). 

Performing the second HSCT from an haplo donor did not result in a better outcome than the other donor-type groups. Conversely, a higher incidence of infection-related deaths in the haplo group did reduce the LFS and the OS compared to the other donor-type groups [96]. 

The results of a most updated comparison between second HSCT from an unrelated or haploidentical donor in AML were published in 2021 by the Acute Leukemia Working Party (ALWP) of EBMT. The outcome of 320 patients undergoing second HSCT from a UD was compared to that of 135 haplo-recipients in the same period (2006–2019). The median age was, respectively, 46 and 44 years old. The survival analysis did not show a difference between the two groups. The two-year OS and LFS were, respectively 31% vs. 29% (*p* = 0.57) and 25% vs. 29% (*p* = 0.73). NRM was 26% vs. 27% (*p* = 0.53). A longer OS was associated again with relapse later than one year after the first transplant (*p* < 0.0001) and with CR-status at the second transplant (*p* = 0.02) [97]. 

The ALWP published the results of a survey on the outcome of a third allogeneic HSCT in 45 acute leukemia patients who relapsed after two previous HSCT. The period covered by the study was between 2001 and 2018, and the median age was 37 (12–71). At the time of third HSCT, 11/45 were in CR while the others had active disease. Twenty-five patients received the third HSCT from a donor different from the second and 30% had at least two donors. The one-year relapse rate was 47%, NRM was 42%. PFS and OS were, respectively, 11% and 20%. In the multivariate analysis, patients who changed donors at least once in their history had a better PFS than the patients who received all the three transplants from the same donor (1-year PFS 17% vs. 0%—*p* = 0.007). Karnofsky PS > 80% and having received the third HSCT from an unrelated donor and after 2010 were correlated with a better OS and PFS [98]. 

To compare the outcome of the second HSCT with that of a DLI-based intervention, the ALWP conducted an observational retrospective study in a cohort of AML patients relapsing after HSCT and treated with a second HSCT (*n* = 137) or DLIs (*n* = 281). The study period covered 23 years between 1992 and 2015. The median age was 43 and 49 years old, respectively, in the second HSCT group and the DLIs group. 

Among the two groups, there were some main differences. In the second HSCT group, with respect to the DLIs group, the percentage of patients in CR at the intervention was significantly higher (38% vs. 18%; *p* < 0.001), relapse occurred at a later time from previous HSCT (348 vs. 211 days; *p* = 0.004) and the median age was significantly younger (43 vs. 49; *p* < 0.001). The donor type was well balanced among groups (MRD and UD were, respectively, 55% and 43% in the second HSCT group vs. 53% and 46% in the DLIs group; *p* = 0.1). In the second HSCT group, 73/137 patients (60%) did receive the transplant from the same donor of the previous HSCT. 

The survival was analyzed by stratifying the groups on time to relapse from first HSCT and remission status at intervention. No significant difference was found between the two groups of intervention. 

Two-year OS in the second HSCT group and in the DLIs-group was, respectively, 11% and 9% in patients relapsing within 6 months (*p* = 0.86) and 36% and 37% in patients relapsing later than 6 months from first HSCT (*p* = 0.53).

Two-year OS in the second HSCT group and in the DLIs-group was, respectively, 35% and 51% (*p* = 0.22) and 20% and 19% (*p* = 0.59) in patients in CR and not in CR at intervention.

Two-year NRM was 26% and 9% for the second HSCT group and the DLIs group (*p* < 0.0001). 

Predictive of better OS were: CR at intervention (*p* < 0.001), relapse later than 6 months from prior HSCT (*p* < 0.001) and prior cGVHD (*p* = 0.02). 

No apparent difference emerged from this study in OS between a DLIs-based intervention and a second HSCT-based intervention. Moreover, NRM in the second HSCT group was significantly higher than in the DLIs group [99]. 

The decision to perform a second HSCT for the treatment of relapse after a first HSCT is questionable. The high morbidity and mortality rates associated with the procedure and the suboptimal medical fitness of the patients make this decision very challenging, and it is often taken into consideration only in very selected cases. Moreover, tools at physician disposition to predict the clinical outcome in this setting are not sufficient. Data from the retrospective analysis are heterogeneous and the comorbidity indexes, useful to predict mortality, have been validated in cohorts of patients largely not yet transplanted [100,101,102,103].

When deciding on the choice of a second HSCT from a different donor, the biological rationale supporting this decision should be based on the attempt to give to the patient a strong GVL effect when a previous GVL has failed. Retrospective studies have shown that a second HSCT can rescue about a quarter of the patients but, at the same time, have failed to demonstrate a clear advantage from changing donors. The high risk of NRM associated with a second HSCT could direct the physicians’ choice towards restoring a silenced GVL effect with DLIs rather than changing it with a different donor, especially if the patient has never experienced GVHD before relapse. In this decision, one should be guided by the careful evaluation of the patient’s performance status, age and medical fitness. Moreover, it should be considered that a second HSCT might have more chance of success when it is performed in CR and the relapse occurs later from the first HSCT (almost over than 6 months). 

Table 2 summarizes the results of selected studies on the second/third HSCT.

## 5. Prophylaxis of Relapse of AML after HSCT

Given the high failure rate of the treatments of the overt relapse after transplantation, strategies aiming at preventing it in the immediate post-transplant period appear more promising [104]. In this setting, we can distinguish three categories of intervention:

(1) Maintenance: a continuative therapy starting early post-HSCT (within the first three months from HSCT during which a valid GVL effect did not yet develop) until drug intolerance or progression of the disease.
(2) Consolidation: a limited number of therapy cycles to consolidate the response achieved by the previous intervention.
(3) Pre-emptive therapy: a therapy which is administered when the hematological malignancy is still subclinical and it is detectable only by flow-cytometric or molecular methods, by anticipating the overt relapse.

Both maintenance and consolidation are interventions aimed at the prophylaxis of AML relapse. 

### 5.1. Prophylaxis of AML Relapse after HSCT: DLIs 

As already discussed, the administration of donor’s lymphocytes in the immediate period post-transplant is associated with a high risk of severe aGVHD. Therefore, early DLIs as prophylaxis (prophylactic DLIs: pDLIs) of relapse should be considered only in case of a high risk of relapse. Such as in the case of acute leukemia with a poor-risk cytogenetics profile (e.g., monosomic karyotype or complex karyotype) or harboring high-risk mutations (e.g., FLT3-ITD at high allelic burden, TP53-mutation) and/or not in CR (primary refractory or untreated relapse) at HSCT. 

Schmid and co-workers designed a sequential regimen of purine-containing chemotherapy (FLAMSA: fludarabine, amsacrine and cytarabine) followed by reduced-intensity conditioning (RIC) with cyclophosphamide and “mini”-TBI + ATG [105]. The regimen included the early withdrawal of CyA (within day +90) followed by prophylactic “escalated-dose” DLIs if no GVHD occurred (starting dose: CD3+ 1 × 10^6^/Kg). Seventy-five high-risk AML patients were treated, and twelve received the planned DLIs. Day + 100 and 1-year NRM were 20% and 33%, respectively. Two-year OS and LFS were 40%. Cumulative incidences of grade II–IV aGVHD and cGVHD were, respectively, 49% and 45%. Patients experiencing limited cGVHD and aGVHD limited to the skin had better survival than those without GVHD or those with more severe GVHD, thus suggesting a GVL effect by limited GVHD [105]. 

More recently, authors from Germany reported the results of a retrospective study on 45 high-risk AML or MDS patients (37 with active disease at HSCT) who were transplanted with the FLAMSA-RIC regimen followed by HSCT and pDLIs. All the patients could receive pDLIs. The results were compared with a historical group of similar patients who have undergone the FLAMSA-RIC protocol without pDLIs. Results did favor the pDLIs: 7-year OS was, respectively 78% and 34% (*p* < 0.001); 6-year LFS was: 68% vs. 38% (*p* = 0.004) [106]. 

The EBMT reported the long-term results of a survey on 318 patients affected with acute leukemia (AML: 78%, ALL: 22%) who underwent prophylactic (*n* = 126) or pre-emptive (*n* = 192) DLIs between 2001 and 2010. Five-year LFS and OS of prophylactic DLIs were 62% and 68%, respectively. Five-year LFS and OS of pre-emptive DLIs were, respectively, 47% and 51% when used in the treatment of minimal-residual disease and 57% and 63% when used in the treatment of mixed chimerism. Five-year NRM in all the settings was 10%. The cumulative incidence of aGVHD and cGVHD was 12% and 30%, respectively. From the multivariate analysis, the risk factors of GVHD were age over 60 years old (*p* = 0.04), transplantation beyond 1st CR (*p* = 0.003), shorter interval between HSCT and DLI (*p* = 0.018) and history of acute GVHD before DLI (*p* = 0.036). In order to evaluate the efficacy and toxicity of the DLIs alone, the patients that have received additional treatments (TKIs, HMAs, chemotherapy) prior to DLIs were excluded from the analysis. For multivariate analysis, only the patients receiving one DLI were considered [107]. 

The potential risks of DLI include pancytopenia and infections in addition to GVHD. In order to reduce the toxicity of DLI, authors have introduced “modified-DLI” (G-CSF-mobilized peripheral blood hematopoietic stem cells—GPBSC) instead of unstimulated DLI [108]. Moreover, immunosuppressive agents (CyA and methotrexate) given after stimulated-DLI have been used to prevent GVHD [109]. This approach may be helpful when the risk of GVHD is exceptionally high, such as when prophylactic DLIs are administered within six months from HSCT. 

Researchers from Guangzhou University investigated the use of stimulated-pDLIs [110]. They assessed prospectively 153 refractory AML patients who underwent HSCT from MRD (55%), UD (27%) and haploidentical (18%) donors by using a sequential regimen of fludarabine-ARA-C chemotherapy followed by RIC conditioning. cyclosporine A was early withdrawn within +90 and G-CSF-mobilized pDLIs were scheduled on day + 60. Among 149 patients, 80 received pDLIs with a median CD3+ dose of 2 × 10^7^/Kg and MNCs of 1 × 10^8^/Kg. The outcome was compared to that of a historical group of 48 high-risk AML patients who did not receive pDLIs after HSCT. Five-year CIR, DFS and OS were better in the pDLIs-group (respectively: CIR 22 and 36%; *p* = 0.04. DFS: 57% and 44%; *p* = 0.02. OS: 58% and 47%; *p* = 0.03). One-year cumulative incidence of aGVHD and cGVHD were 62% (14% gr III–IV aGVHD) and 61% (extensive cGVHD 21%), respectively. 

In the multivariate analysis, predictors of longer OS and DFS were the use of pDLIs (*p* = 0.04) and cGVHD (*p* = 0.03). Additionally, the percentage of BM blasts on day 0 < or ≥ 3% did result predictive of survival (*p* = 0.003) [110]. 

Rui Zhang and co-workers explored the use of modified pDLIs by combining them with decitabine. They conducted a prospective, single-arm study in 28 patients affected by high-risk genetic AML (FLT3-ITD, TP53mut, ASXL1mut, DNMT3A, TET-2). Donors were MRD and haploidentical, respectively for 10 and 18 transplants. The conditioning was a modified BU-Cy (busulphan, carmustine, cytarabine and cyclophosphamide) regimen followed by a single G-CSF-mobilized pDLI (target dose: CD3+ 2 × 10^7^/Kg) between day +30 and +60 (in MRD-recipients) and +60 and +90 in haploidentical (pDLIs were done during the immunosuppression with CyA through a concentration of 150–250 ng/mL). Ciclosporine A, methotrexate and mycophenolate mofetil (MMF) were given as GVHD prophylaxis and ATG was added in transplantations from haploidentical and from MRD over 40 years old. In patients co-expressing FLT3-ITD with a high-risk genetic mutation, pDLI was preceded by a single cycle of decitabine 10 mg/m^2^ for five days. Three-year CIR, RFS, and OS were 26%, 48% and 48%. Cumulative incidence of aGVHD and cGVHD were 25.8% and 21%, and NRM was 25%. Using multivariate analysis, aGVHD and relapse after pDLIs resulted significant for shorter OS (*p* = 0.016 and *p* = 0.003) [111]. 

Other authors have retrospectively compared the outcomes of stimulated-pDLIs in a group of 21 haploidentical HSCT with that of a group of 13 MRD-HSCT. Among haploidentical recipients, the 100-day post-DLI incidence of aGVHD and 1-year NRM were higher than among MRD-recipients (grade II–IV aGVHD post-DLI: 60% vs. 30%; *p* = 0.05. one-year NRM: 27.9% vs. 0; *p* = 0.06). However, the use of pDLIs in haploidentical recipients resulted in a better 1-year relapse rate with respect to a small historical control group of haplo-recipients (n = 8) without pDLI (28% vs. 62% respectively. *p* = 0.03). Haploidentical pDLIs effectively prevented relapse but at the expense of a higher risk of aGVHD and NRM than MRD pDLIs [112].

Prophylactic DLIs represent a strategy aimed at anticipating the GVL effect and appear to be efficacious in preventing relapse in high-risk AML with respect to historical controls that did not receive pDLIs. However, to achieve their scope pDLIs should be administered early after transplant and, given the high risk of severe acute GVHD, caution should be taken in the choice of the most appropriate dose and escalation protocol to be used. A high risk of mortality has been described, particularly following haploidentical pDLIs, and this risk could overcome their potential effect in preventing disease. The ALWP recommended a dose of pre-emptive Haplo-DLI of CD3+ 1 × 10^5^/Kg [63]. In order to prevent the risks of pDLIs, the use of G-CSF primed DLIs followed by a short course of immunosuppression as GVHD prophylaxis may be considered. 

### 5.2. The Hypomethylating Agents in Prophylaxis after HSCT

The immune-modulating activity of the HMAs and their effect on the tumor microenvironment and on antigen presentation have already been discussed. Herein, we describe the results of the clinical trials investigating the use of HMAs in the prophylaxis of relapse after HSCT. 

From 2010 up today, several prospective studies have been conducted evaluating the feasibility and the effectiveness of the HMAs in preventing disease relapse after HSCT. 

Researchers from MD Anderson Cancer Center (MDACC) first studied 5′-azacytidine (5′-AZA) as maintenance therapy post-HSCT. 

De Lima and co-workers conducted a phase I dose-finding study in 45 patients undergoing HSCT for high-risk AML/MDS and treated with 5′-AZA as maintenance (67% had active disease at HSCT). Five dose levels of 5′-AZA were evaluated (from 8 mg/m^2^ to 40 mg/m^2^ for 5 days in a 25-day cycle). Maintenance started from day +40 up to six cycles of 5′-AZA. At a median follow-up of 20 months, NRM was 9% and the incidence of relapse was 53%. The best-tolerated dose was 32 mg/m^2^ for 5 days in a 25-day cycle [113].

Other researchers at the Missouri University performed a phase I study on the use of decitabine as maintenance in 24 high-risk AML/MDS patients after HSCT by starting between day + 50 and +100. Four dose levels were evaluated: from 5 mg/m^2^ to 15 mg/m^2^ for five days in a 6-week cycle up to a maximum of eight cycles. After a median follow-up of 16.7 months, CIR was 28%, and 2-year OS was 56%. No MTD was observed. The authors concluded that 10 mg/m^2^ for five days in a 6-week cycle might be the most appropriate schedule because most hematological toxicities occurred at the superior level of 15 mg/m^2^ [114].

In the RICAZA trial, the authors evaluated the feasibility of 5′-AZA as maintenance after RIC HSCT in 37 AML patients. The median age was 60 years old (40–71), and conditioning was a RIC regimen (Flu-Mel: fludarabine and melphalan) with in vivo T-depletion with alemtuzumab. 5′-AZA was administered at 36 mg/m^2^ for five days in a 28-day cycle. Maintenance started on a median of 54 days after HSCT and was scheduled for 12 months. Thirty-one patients completed at least three cycles, and sixteen completed ten cycles. One hundred days and 1-year mortality were 0 and 8%, respectively. The 2-year RFS and OS were both 49%. The authors found, also, that a significant antitumor response induced by T-CD8+ cells correlated with an improvement in RFS (*p* = 0.02) [78].

Recently, an oral formulation of azacytidine (CC-486) has demonstrated a significant advantage over placebo in a phase III study in maintenance after intensive chemotherapy in patients in CR not eligible for HSCT [115]. 

MDACC conducted a phase I/II study on oral maintenance with CC-486 in patients in CR after HSCT. The study enrolled 30 patients (26: AML, MDS: 4) receiving transplantation from an MRD (10) or an unrelated (20) donor. The primary end-point was the MTD of CC-486, and four dose levels were investigated in a 3 + 3 dose-escalation design: 200 mg/day and 300 mg/day for seven days, 150 mg and 200 mg for 14 days in a 28-day cycle. The maintenance started in the median at day +81. No MTD was observed, and the fourth dose level of 200 mg/day for 14 days was utilized for the second part of the study to assess the safety and tolerability. The one-year relapse rate was 21%. The median OS was not reached. Only one patient experienced severe aGVHD (grade III); nine patients (30%) had cGVHD (three severe, six mild/moderate). 

Twenty-two patients experienced at least one grade 3–4 adverse event (AE). The most common AEs were gastrointestinal and hematologic [116]. 

French researchers conducted a phase II study on prophylaxis with a combination of low-dose 5′-AZA and DLIs. The study enrolled 20 AML patients with poor-risk cytogenetic or active disease at transplant and 10 MDS patients with intermediate-2 or higher IPSS risk scores. The prophylaxis with 5′-AZA began in median 66 days after HSCT (range 38–93 days) while on treatment with CyA, and the dosage was 32 mg/m^2^ for five days in a 28-day cycle. DLIs were scheduled at escalating doses after cycles 3, 5 and 7. Only ten patients completed the scheduled 12 cycles, and 17 patients could receive at least one DLI. The results were compared with a historical control group of patients with similar characteristics but not receiving 5′-AZA or DLIs. The study group’s relapse was inferior although not statistically significant (2-year CIR: 27% vs. 41%; *p* = 0.2) [117].

In 2020, two large randomized trials investigating the role of HMAs in maintenance have been published. 

In the US, a multicentric study randomly assigned (1:1) 187 high-risk adult AML or MDS patients to receive 5′-AZA 32 mg/m^2^ for five days in a 28-day cycle up to 12 cycles or observation. The prognostic risk was based on cytogenetics (complex karyotype or chromosome 5 or 7 abnormalities), primary induction failure or relapsed disease prior to transplantation, therapy-related AML or MDS, biphenotypic AL. Of note, the study was characterized by a high screening failure rate (among 561 screened patients, 362 did not enter into the study because of failing eligibility or lack of interest). 

The median time to start treatment was day +62 (42–100). Among the 93 patients assigned to maintenance, only 24 (27%) could complete the planned 12 cycles of 5′-AZA. Sixty-three patients did exit from the study mainly for disease relapse (*n* = 29), infections (*n* = 7), toxicity (*n* = 11), GVHD (n = 2) and patients’ decision or logistical reasons (*n* = 14). In this study, the maintenance with 5′-AZA failed to improve the RFS compared to the control arm. Median RFS were 2 and 1.8 years, respectively (*p* = 0.43). The only factor associated with better RFS was CR at HSCT instead of active disease (*p* = 0.007) [118]. This study failed to demonstrate a clear advantage from the maintenance with azacitidine over observation. However, some criticisms have been highlighted: the high rate of screening failure and the slow accrual (from 2009 to 2017), which caused the trial to close prematurely [119]. 

In China, a multicentric randomized study investigated the combination of recombinant-human granulocyte-colony stimulating factor (rhG-CSF) with decitabine in the maintenance post-HSCT. Rh-GCSF may adjuvate the cyclin-dependent activity of decitabine by promoting the cell-cycle entry in the leukemic blasts. 

The study randomized 1:1 the patients to receive G-CSF 100 mg/m^2^ from day 0 to 5 and decitabine 5 mg/m^2^ iv from day 1 to 5 in a six-week cycle up to a maximum of 6 cycles (G-DEC group) or observation only after HSCT (controls). 

From April 2016 to January 2017, 204 high-risk adult AML patients were enrolled. High-risk was defined as poor-risk cytogenetics, primary refractory, relapsed or secondary AML. The post-transplant MRD was positive in 24% of the G-DEC group and 28% of the controls. 

The 2-year CIR was, respectively, 15% and 38% in the G-DEC and in the control group (*p* < 0.001). The superiority of the G-DEC arm was also maintained when stratifying by MRD status. In MRD-positive patients 2-year CIR was 34% in the G-DEC and 59% in the control arm (*p* = 0.05) while in MRD-negative it was 6% and 31%, respectively (*p* < 0.01). 

The authors also observed a significant increase in T-CD8+ cells, NK and T-regs after the second and third cycle of G-DEC and in a multivariate model, increased NK cells were associated with a lower relapse rate [120].

In conclusion, the use of HMAs in the prophylaxis of relapse after HSCT appears to be feasible. Given their role as immune-modulating agents, and in order to prevent their hematological toxicity, the maintenance dose may be lower than the treatment dose. The doses of 32 mg/m^2^/day for 5 days for 5′-AZA and of 10 mg/m^2^/day for 5 days for decitabine demonstrated an acceptable toxicity profile from phase I studies [113,114]. The recently approved oral formulation of CC-486 has been tested in the post-HSCT setting with promising results in terms of efficacy and manageability [116]. The large multicenter phase III study on 5′-AZA in maintenance post-HSCT did not provide convincing results [118] but doubts have been raised regarding possible selection bias [119]. Conversely, a large, randomized trial on low-dose decitabine in maintenance did demonstrate a clear advantage over control in preventing relapse. In this study, the effect of decitabine has been used in combination with the priming effect of the rhG-CSF [120]. 

Table 3 summarizes the results of selected studies on HMAs in the prophylaxis of relapse after HSCT.

### 5.3. FLT3-Inhibitors in Maintenance 

FLT3, a tyrosine-kinase receptor, is one of the most frequently mutated genes in AML blasts. Two different abnormalities may occur, internal-tandem duplication (ITD), which is the most common, and point mutations in the tyrosine-kinase domain (TKD). Both result in constitutive activation of the receptor with consequent stimulation of cell proliferation and inhibition of cell differentiation [121]. 

About 25–30% of adult AML patients harbor FLT3-mutations. Given the high relapse rate FLT3-mutation confers a poor prognosis [122]. 

Hematopoietic transplantation is the only curative option but even after HSCT a high percentage of patients still relapse [9].

Several FLT3-inhibitors (pertaining to the class of tyrosine-kinase inhibitors: TKIs) have been developed. TKIs interact with the ATP-binding site in FLT3, hindering the receptor activation. 

According to their specificity and potency, TKIs can be divided into two main categories: first-generation (sorafenib, midostaurin) and second-generation TKIs (gilteritinib, quizartinib, crenolanib) [123]. The latter have less off-target effects with a consistent reduction of serious complications related to drug administration. 

FLT3-inhibitors demonstrated effectiveness in the setting of relapsed/refractory disease and, together with induction chemotherapy, in newly-diagnosed patients [124,125,126]. 

Midostaurin is used in the induction, consolidation, and maintenance therapy for newly diagnosed FLT3 mutated AML [127]. Indeed, its use has been demonstrated to increase overall survival compared to chemotherapy alone [128]. 

The RADIUS trial is a phase II, randomized, open-label trial aiming at investigating the effectiveness of midostaurin in the post-HSCT maintenance of FLT3-mutated AML. 

The patients were randomly 1:1 assigned to receive, starting from day +28–60, oral midostaurin 100 mg/day in a 4-week cycle up to 12 cycles vs. standard of care (SOC). SOC was at the physician’s discretion, but other FLT3-inhibitors were excluded. The primary end-point was RFS, but the study was not powered to detect a statistical significance between the two arms because it was intended as an exploratory study. 

Sixty patients were enrolled (30 per arm), but only half of them (16 in the study arm and 14 in the control arm) could complete the scheduled 12 cycles of therapy. Adverse events occurred in 100% of the patients in the midostaurin arm and in 87% in the control arm. The most common toxicity of the midostaurin arm was gastrointestinal (emesis and diarrhea). 

The estimated 18-month RFS was, respectively, 89% vs. 76% in the midostaurin arm and in the control arm (*p* = 0.27). The study comprised a pharmacokinetic assay by measuring the FLT3-inhibition in plasma. The highest level of FLT3-inhibition was reached during the first two cycles of midostaurin, while steady-state levels were reached after cycle 4. 

A high level of FLT3-inhibition (i.e., FLT-3 phosphorylation inferior to the 70% of the baseline at day 1 of the third cycle) was associated with a better RFS, and it was reached in 14 patients. The authors concluded that in patients able to complete the planned treatment, the degree of FLT3-inhibition is high, resulting in better disease control [129]. 

Sorafenib is a multi-kinase inhibitor that in a mouse model has demonstrated the ability to promote a strong GVL effect by inducing the release of IL-15 by tumor cells. The IL-15 released by AML cells induced the activation and expansion of a subset of T-CD8+ cells cytotoxic towards AML cells. In mice, sorafenib synergizes with DLI and in sorafenib/DLI responders was observed an expansion of cytotoxic, long-persistent, anti-leukemic T-CD8+ cells expressing high levels of BCL-2 and low levels of PD-1 [20].

Two randomized studies evaluated the role of sorafenib in the prophylaxis post-HSCT in FLT3-mutated leukemia. 

Li Xuan and co-workers conducted an open-label, phase III study in 202 FLT3-mutated patients who were enrolled between 2015 and 2018 to receive oral sorafenib or not (controls). Sorafenib was started at 30–60 days post-HSCT up to day +180 at a dosage of 400 mg × 2/die. 

With a median follow-up of 21 months, the 1-year CIR was, respectively, 7% and 24% in the sorafenib and in the control group (*p* = 0.001). The incidence of GVHD, infection and hematological toxicity did not differ between the two arms.

In the multivariate analysis, the variables associated with RFS were MRD status at enrollment (*p* = 0.02) and the use of sorafenib (*p* < 0.0001). For a better OS, only sorafenib resulted significant (*p* = 0.007) [7].

In Germany, a multicentric, double-blind study randomized 1:1 eighty-three adults FLT-3-positive AML patients to receive sorafenib as maintenance post-HSCT or placebo (SORMAIN trial). Treatment started between day +60 and +100 until 24 months or until relapse or intolerable toxicity. With a median follow-up of 41 months, the RFS was 85% and 53%, respectively in the sorafenib and in the placebo group (*p* = 0.002). 

The two previous studies provided evidence for the efficacy of sorafenib in reducing relapse of FLT3-mutated AML post-HSCT [6]. 

FLT3-inhibitors represent a new and rapidly expanding field of research. Such agents are very promising also in the post-transplant setting and potential combination strategies (for example with HMAs, checkpoint inhibitors, small-molecule inhibitors) could be investigated in the future to improve their anti-leukemic activity while minimizing the toxicity. Some studies investigating the second generation FLT3-inhibitors in the maintenance of CR after HSCT are ongoing.

The NCT02400255 is a single-arm, phase II study evaluating the role of crenolanib in the maintenance of remission after HSCT in FLT3-mutated AML. The start of treatment is scheduled between days + 45–90 after transplant and the primary endpoint is the 2-year PFS. The patients are stratified according to CR or CRi at HSCT. 

The NCT02997202 is a multicentric, randomized, phase III study investiganting the role of gilteritinib in the maintenance after HSCT in FLT3-ITD mutated patients in first morphologic CR (MORPHO-STUDY). The study is actually active, the estimated number of enrolled patients is 532 and the estimated completion date is april 2025. The study randomizes the patients to receive gilteritinib or placebo between days 30 and 90 after HSCT. The patients are stratified according to conditioning intensity (MAC vs. RIC), time from HSCT to randomization (days 30–60 vs. 61–90) and MRD (positive vs. negative). The primary end-point is the 7-years RFS. 

## 6. Pre-Emptive Therapy

MRD monitoring can identify patients with a higher risk of relapse after HSCT and several intervention methods are available for MRD-positive patients after HSCT [31].

Pre-emptive approaches, initiated at the time of the MRD detection, can avoid overt relapse [8].

As already discussed, the hypomethylating agents 5′-azacitidine (AZA) and decitabine (DAC), have a direct anti-leukemic effect which is independent from a distinct molecular phenotype and also, positively influences the GVL-GVHD balance. Moreover, their toxicity profile is more favorable than conventional chemotherapy [130,131,132,133].

Platzbecker and colleagues tested the pre-emptive therapy with azacitidine at the time of molecular relapse in two prospective trials. 

In an initial proof-of-concept study (RELAZA-1) 20 patients with MDS or AML were treated with up to four cycles of AZA as soon as the CD34+ donor chimerism in peripheral blood dropped below a threshold of 80%, while patients were still in complete remission [134].

Despite an improvement of chimerism (>80%) in half of the patients, this early intervention was able to induce durable remissions only in three (30%) of the responders and did not avoid progression towards overt relapse in the majority of patients. The authors expanded this analysis in a second trial (RELAZA-2) covering 53 patients (24 after HSCT, 29 after conventional chemotherapy), who were monitored by CD34+ donor chimerism or molecular markers such as NPM1 and RUNX1-RUNX1T1.

In the case of MRD positivity, the patients could receive up to 24 cycles of AZA. The study met its primary end-point with 31 patients (58%) free of relapse after 6 months and 19 of 53 patients achieving a major response (36%). With a median follow-up of 13 months after the start of therapy, 1-year RFS was 46% [135].

Besides the pharmacological approaches, cellular interventions are also an option to prevent and treat relapse of AML.

Donor lymphocyte infusions (DLI), as already discussed, are a cellular product of mononuclear cells containing a defined number of donor-derived CD3+ T-cells. The DLI can be obtained either as aliquots from the G-CSF-mobilized PB stem cell product or by an unstimulated leukapheresis of the original donor [135].

Some retrospective analyses and a limited number of prospective studies have reported on the use of DLI as a prophylactic approach and showed that DLI administration in patients with increasing mixed chimerism (MC) decreased the relapse rate and favorably affected outcomes [136,137,138,139,140].

Krishnamurthy and co-workers reported the outcome of 113 AML/MDS patients treated with DLI as pre-emptive (62 patients) or therapeutic (51 patients) intervention after HSCT conditioned with a reduced-intensity regimen (RIC) and T-depleted in vivo by Alemtuzumab (99) or ATG (14). Pre-emptive DLIs were given to restore a persistent or increasing mixed donor-recipient chimerism. All DLIs were administered after the withdrawal of cyclosporine A. The DLIs administered as pre-emptive and as therapeutic intervention resulted, respectively in an OS of 80% and of 40%.

In the pre-emptive group, the outcome did not differ according to the class of risk or the duration of the first remission. In the therapeutic DLIs group, 70% of patients had received adjuvant chemotherapy and the outcome was different according to the cytogenetic risk and the duration of remission. The survival was inferior in patients relapsing earlier than 6 months from HSCT (OS 11% vs. 51%, respectively; *p* = 0.008) and in patients belonging to the poor risk than to the intermediate/low-risk cytogenetic class (5-year OS 0%, 33% and 55% respectively; *p* < 0.01) [138].

In a prospective multicenter study in 71 children with AML and MC after HSCT, immunosuppression was stopped and DLI was administered if no GvHD occurred after 3–4 weeks. To repeat the DLI at an increased dose was allowed if MC persisted in the absence of GVHD. Thirteen out of twenty children with MC received DLIs. The EFS was, respectively, 80% and 30% for patients with full donor chimerism and mixed chimerism. Patients with MC who received DLIs had an EFS of 46%, while 100% of MC patients relapsed without DLIs (*p* = 0.009) [139].

Dominietto and co-workers reported the results of a retrospective analysis of pre-emptive DLIs administration in a group of 80 patients with acute leukemia (36 AML, 44 ALL) after HSCT. The MRD monitoring was performed on monthly BM samples using RT-PCR for detection of WT1 transcripts. All the patients with measurable MRD, with an available donor and without evidence of GVHD, received pDLIs. The cumulative incidence of relapse was 16% in MRD-negative patients, compared with 6% of MRD-positive patients treated with DLI and 63% of MRD-positive patients without DLI (*p* = < 0.001) [140].

In the case of MRD positivity detection following HSCT, we suggest confirming the result within 1–2 weeks with a second bone marrow evaluation. If the result is confirmed, we would promptly interrupt immunosuppressive therapy, clearly only in the absence of GVHD, and we would start a pre-emptive therapy as soon as possible. The best approach, in our opinion, is based on the combination of DLI infusions and novel therapies, usually hypomethylating agents or targeted therapy (the latter if the disease harbors a specific molecular target, such as FLT-3). DLI infusion must be avoided in case of active GVHD. Indeed, in a patient presenting with active GVHD and an MRD positivity, the disease might have become resistant to the GvL effect through immune-evasion mechanisms. In this case, the DLI would be useless and harmful to the patient, and the initial intervention for MRD positivity would be based only on pharmacological agents (e.g., HMAs +/- venetoclax or FLT3-inhibitors). Once a pre-emptive therapy is started, close monitoring of MRD status is required, in order to modify the therapeutical approach in case of the evolution of the disease. If possible, both immunophenotyping and molecular analysis should be used to detect MRD. The cut-off and the time-points for MRD-monitoring are that recommended by the ELN guidelines [36]. In the absence of a LAIP or of a specific MRD molecular marker, the increasing mixed chimerism might be considered as predictive of relapse, and, especially in high-risk diseases, might justify the start of pre-emptive therapy. It would be better if the chimerism is performed on CD34+ cells of BM [141], although this is outside of daily practice for most laboratories. 

## 7. Immunologic Therapies

### 7.1. Novel Cellular-Based Interventions

Immunotherapy is one of the most interesting approaches for the treatment of acute myeloid leukemia [142,143]. Hematopoietic transplant is an immunotherapy in which the donor-derived T-cells play a pivotal role in controlling the relapse of AML throughout a polyclonal immune response. Such graft-versus-leukemia effect is at the expense of an immune response against the healthy tissues (GVHD). New strategies based on antitumor T-cells are necessary to achieve an anti-leukemic effect while avoiding GVHD. Down-regulation of MHC-molecules is one of the most frequent mechanisms responsible for immune escape [22,144]. This phenomenon can be overcome by employing a non-MHC-dependent T-cell’s mechanism of action.

Chimeric antigen T-cell receptor (CAR-T) therapy is a form of adoptive immunotherapy in which autologous or allogenic T-lymphocytes receive a genetic manipulation to express a recombinant T-cell receptor capable of recognizing the tumor-associated-antigens. The chimeric-antigen-receptor acts in a non-MHC-dependent manner and may induce activation, proliferation, and the effector function of the modified T-cells [145]. 

CAR-T therapy has been approved for the treatment of B-cell malignancies [146,147,148].

CAR-T therapy can abrogate MRD and provide a high rate of complete remission and a long progression-free survival [146].

The main obstacles in developing this immunologic therapy in the treatment of AML are represented by difficulties in the selection of the target antigens and by the leukemic microenvironment, which acts by abrogating the cytotoxic properties of the CAR-T. 

The ideal properties of the target antigens should be [149]:

(1) Expression only by the tumor and by the leukemia stem cells (LSC) that are responsible for relapse.
(2) Absent expression by the normal hematopoietic tissue and by the extra-medullary healthy tissues.

The tumoral population of AML is composed of a proliferative compartment and of an LSC compartment. The latter is responsible for the regenerative potential. LSC are capable of initiating and maintaining the disease. Targeting both the cell-compartments of AML is of primary importance to achieve a prolonged remission. Moreover, tumor antigens are variably expressed, and this allows for relapse after immunotherapy when this is directed toward a single antigen. This issue can be overcome by the use of immunotherapy toward multiple target antigens [150]. 

The antigens shared in common by tumoral and healthy tissues are an obstacle to immunological-based interventions in treating AML. In the early trials, the effect of CAR-T on the normal hematopoietic tissue induced prolonged cytopenia [151]. 

To date a relatively limited number of AML patients treated with CAR-T has been reported and with a low rate of disease response [149]. 

The current clinical trials on CAR-T and NK in AML are based on constructs directed towards CD33, CD123, CLL-1, NKG2D, CD44v6 and C-type lectin-like molecule-1 (CLL-1). The latter is a transmembrane glycoprotein that is expressed by AML cells in 80% of adult patients and in most pediatric patients. The expression of CLL-1 on normal hematopoietic tissues is low. It is one of the most promising antigens as a target candidate for CAR-T therapy. Encouraging results arise from one study in four pediatric patients. Of them, 3 achieved a complete response with 2 MRD responses [152]. Another approach to avoid the antigens shared in common is targeting the tumor-associated-variant isoforms. CD44v is a variant isoform expressed on 60% of AML. A study investigating in vitro and in vivo efficacy of CAR-T directed towards CD44v is ongoing [153]. At actual time CAR-T therapy is an attractive immunotherapy for acute myeloid leukemia but it is still in development. Moreover, currently the results from the clinical trials, despite the promising activity from the pre-clinical studies, are unsatisfactory with respect to disease control. Further studies are necessary to improve the CAR construct, the choice of the target and carrier cells and the modulation of the tumor microenvironment.

Most of the target antigens are expressed on blast cells and also on normal hematopoietic and non-hematopoietic cells. As an example, CD7+ is expressed on T-cell, CD33+ on Kupffer-cells, CD 123 on endothelial cell, CD44v6 on keratinocytes and CCL1 on lung and gastrointestinal cells. The “on/off target” toxicities of the CAR-T are less tolerable than B-cell aplasia and can be lethal upon prolonged exposure. The CAR-T therapy in AML should be considered a preparatory intervention to HSCT rather than a single therapy [143]. 

Table 4 depicts the currently available data. Most of the studies have shown a low rate of complete response with most of the patients dying from progression of the disease [154,155,156,157,158,159,160,161,162,163]. A recent study from the MDACC highlighted some of the difficulties arising from applying this intervention to the treatment of AML. In this phase I study ten patients were enrolled, apheresis was collected from eight patients, four could have the CD33-CAR-T product, one patient died before receiving it and, finally only three patients could receive the therapy. All patients died from progression of the disease. This study was closed, and the platform used for the construction of CAR was replaced by another platform considered more efficient [163].

### 7.2. Immunotherapeutic Strategies

Recently, novel immunotherapeutic strategies entered the therapeutic scenario of AML, including immune checkpoint inhibitors, monoclonal antibodies and vaccination. 

### 7.3. Immune Checkpoint Inhibitors

Ipililumab is a monoclonal antibody that activates T-cells by blocking CTLA4 and it can restore the GVL effect in AML relapse following HSCT. In a phase I trial, among 22 patients with relapsed AML following allogeneic HSCT, five achieved a complete response (including four with extramedullary disease), two had a partial response and in six patients a reduction of the tumor load was obtained. This although immune-mediated toxic effects and GVHD occurred [166]. It has been shown that the expression of PD-L1, PD-L2, PD-1 and CTLA4 was up-regulated in patients undergoing treatment with HMAs; the up-regulation was higher in patients resistant to therapy compared with patients who achieved a response to treatment [167]. The up-regulation of immune-checkpoint molecules seems to be a mechanism of resistance to HMAs and supports the use of combination therapy with immune checkpoint inhibitors. Ipililumab is being evaluated in combination with decitabine in post-HSCT and transplant-naïve patients with relapsed/refractory myelodysplastic syndromes and AML [168]. Nivolumab is a monoclonal antibody, which binds to the PD-1 receptor and blocks its interaction with its ligand PD-L1, preventing T-cell inhibition. A phase I/II study involving 70 high-risk relapsed/refractory AML patients treated with the combination nivolumab–azacitidine showed an ORR of 33%, including 15 complete remission/complete remission with insufficient recovery of counts (CR/CRi). The ORR was 58% and 22%, in HMA-naïve and HMA-pretreated patients, respectively [24]. Additionally, pembrolizumab, another monoclonal antibody targeting PD-1 receptor, has been evaluated in combination with HMAs with similar results [169,170]. 

Magrolimab is a macrophage checkpoint inhibitor inducing tumor phagocytosis by blocking CD47. The FDA recently assigned breakthrough designation to magrolimab, in combination with azacitidine, for the treatment of adult patients with newly diagnosed MDS. The FDA approval is based on the results of a phase Ib trial in which the combination magrolimab-azacitidine was used in newly diagnosed MDS and AML patients ineligible for intensive chemotherapy. This phase Ib trial evaluated 68 patients (MDS = 39; AML = 29), including 12 *TP53^mut^* patients, and the ORR was, respectively 91%, in MDS patients and 64% in AML. Particularly, ORR was 75% in *TP53^mut^* patients. 

### 7.4. Antibody-Based Therapy

Monoclonal antibodies in AML include toxin-conjugated antibodies targeting CD33 and CD123, anti-CD33 and anti-CD45 radio-conjugated antibodies and multiple bispecific antibodies. Gemtuzumab-ozogamicin (GO), an anti-CD33 antibody conjugated with calicheamicin, is the first antibody-based therapy approved for the treatment of AML in combination with induction chemotherapy. GO induced a significant survival benefit only in the good-risk cytogenetics patients [171,172]. In the relapsed/refractory setting GO induced a complete remission rate of 26% as monotherapy and 24% in association with azacitidine [173,174]. The role of GO in the treatment of post-HSCT relapse has not been evaluated although few positive experiences regarding the use of GO in treating extramedullary relapse post allogeneic HSCT have been reported [175,176]. Furthermore, the role of GO has been evaluated in association with hypomethilating agents as a maintenance therapy in high-risk patients undergoing HSCT [177]. 

CSL360 is a recombinant, chimeric IgG1, anti-CD123 monoclonal antibody that neutralizes IL-3. In a phase I study involving 40 patients with advanced AML (seven who had undergone HSCT), five dose levels of CSL360 were administered at doses of 0.1–10.0 mg/kg. Despite complete saturation and down-regulation of CD123, indicating successful IL-3 signal blockade, only two patients responded. However, it is worth noting both responders had undergone prior HSCT or CSL360 [15]. 

### 7.5. Vaccines

The therapeutic vaccines in AML aim at inducing a cellular immune response against tumoral cells. The main categories of the anti-cancer vaccines are antigen-specific vaccines, whole-tumor cell vaccines and dendritic cell (DC)-based vaccines [178]. Leukemia-associated antigens include Wilms’ tumor 1 (WT1) antigen, proteinase (PR)-1 and -3, preferentially expressed antigen of melanoma (PRAME) and receptor for hyaluronic acid-mediated motility (RHAMM). Limitations to this approach are the potential immune escape through the down-regulation of the target antigens and the clonal heterogeneity, which might be responsible for the lack of susceptibility by a sub-clone of tumoral cells. Another approach is the whole-tumor cell vaccines that can better capture tumor heterogeneity. An example is the GVAX platform, in which patient-derived tumor cells are transduced with an adenoviral vector expressing GM-CSF to induce an immune response [77]. However, both approaches are dependent on an effective platform of antigen presentation and the use of DC vaccines can overcome this limitation. 

Ho et al. performed vaccination in refractory myelodysplastic syndrome/AML patients with GVAX early after post-HSCT (+30 to +45 days) [77]. Despite the use of immunosuppressive agents, vaccination induced a significant immune reaction with durable responses without a significant increase in the GVHD rate. 

Maeda et al. reported the results of a phase I clinical trial on WT1 vaccination in nine post-HSCT patients who were at high risk of relapse or had already relapsed [179]. Three AML patients, who had undergone HSCT in non-CR, started WT1 vaccine in CR on days 141, 76 and 93 post-HSCT and have remained in CR for 1038, 973 and 662 days. Six patients started WT1 vaccination in non-CR and two of them became CR after WT1 vaccination. 

## 8. Novel Targeted Agents in Development

### 8.1. Small-Molecule Inhibitors 

Small-molecule inhibitors (SMIs) are emerging drugs in the therapeutic landscape of AML relapsing after HSCT. The main targets of this class of drugs are represented by molecules that are crucial for the survival and proliferation of leukemic cells, such as B-cell lymphoma-2 antiapoptotic protein (BCL-2), FMS-related tyrosine kinase 3 (FLT-3) and proteins involved in Hedgehog signaling [121,180,181]. 

The FLT3-inhibitors have already been discussed above. 

Targeting the anti-apoptotic protein Bcl-2 is another promising therapeutic approach for the treatment of relapsed AML after allo-SCT. Indeed, the combination of venetoclax with azacytidine and DLI, in this setting of patients, induced 26.9% of CRi and 34.6% of PR. Looking at the event-free survival (EFS) and OS they were 120 and 284.5 days respectively [90].

The RAS/RAF/MEK/ERK pathway is often dysregulated in AML leading to cell survival and resistance to treatments [182]. To counteract this pathological signaling, trametinib, an anti-MEK molecule has been developed. An ongoing phase II trial, NCT04487106, is evaluating the association of azacytidine with two SMIs, venetoclax and trametinib, for the treatment of R/R AML. 

Another pathway, which is crucial for the survival of the leukemic blasts and their proliferation is represented by the Hedgehog pathway. Recently, glasdegib, a novel small-molecule inhibitor targeting the key regulator of the Hedgehog pathway, has been approved for newly diagnosed unfit AML patients [183]. In particular, glasdegib is able to inhibit smo, a protein that activates the Hedgehog pathway [184]. 

Emerging studies are now evaluating the administration of this SMI in relapsed AML. Zucenka et al. have reported the use of glasdegib combined with low-dose cytarabine in 31 patients affected by R/R AML, 9 (29%) of whom relapsing early after HSCT. The median overall survival was 10.4 months and interestingly, an univariate analysis revealed that previous HSCT and venetoclax exposure did not significantly influence the survival [185]. 

The NCT01841333 is an open-label, phase II trial evaluating the role of PF-04449913 (glasdegib) in the maintenance after allogeneic HSCT. The dosage is 100 mg/day from day +80 for up to one-year post-transplant or until unacceptable toxicity or relapse. 

The NCT04655391 is a phase Ib study evaluating the best-tolerated dose and the effectiveness of glasdegib with various other novel drugs in AML relapsing after HSCT. The study is ongoing and its estimated completion date is December 2023. The purpose is to assign the patients to one of five arms of treatment. The assignment occurs after a molecular diagnosis has been made. In each arm the patient is assigned to a molecular segment or to a treatment segment in which they will receive, respectively, glasdegib alone or in combination with various molecular-targeted agents: gilteritinib, bosutinib (a BCR-ABL-inhibitor), ivosidenib, enasidenib or venetoclax and decitabine (the latter two used in combination). Primary end-points are the proportion of patients who have a successful molecular diagnosis (e.g., a successful molecular sequencing) and the proportion of patients who are assigned to a treatment arm.

The Gruppo Italiano Malattie Ematologiche dell’Adulto (GIMEMA) is the sponsor of a phase III trial (NCT04168502) assessing the role of gemtuzumab–ozogamycin added to the standard induction and consolidation chemotherapy in reducing the MRD levels in de novo, favorable/intermediate-risk AML. The study also provides post-transplant maintenance where patients are randomized to receive glasdegib 100 mg/day orally or observation. Primary end-points are levels of pre-transplant MRD and DFS up to 2.5 years. The study started in September 2020 and the estimated completion date is on April 2027. 

An interestingly SMI against E-selectine, known as uproleselan, has been tested in patients with R/R leukemia. The results from the phase I/II study, NCT02306291, demonstrated that the addition of uproleselan to standard chemotherapy induces a remission rate of 41% with a median OS of 8.8 months [186]. A phase III clinical trial (NCT05054543) is now ongoing to confirm the efficacy of uproleselan in R/R AML patients, including the ones who relapsed after HSCT. 

The role of the SMIs in the post-transplant setting is under investigation and it remains to be better evaluated.

### 8.2. Histone-Deacetylase Inhibitors

Aberrant epigenetic modifications of chromatin such as DNA-hypermethylation and histone acetylation are involved in the maintenance of leukemia [187]. Besides HMAs, the histone-deacetylase inhibitors (HDACi) may act by restoring epigenetic deregulation of chromatin in leukemic cells. 

Panobinostat is an HDACi that has been investigated in a phase I/II study in 42 high-risk patients (AML: 37. MDS: 5) as a maintenance post-HSCT (PANOBEST study). The primary objective of the study was to find the MTD and RFS. 

Best-tolerated doses were 20 mg × 3/day every week or 30 mg × 3/day every other week in a 28-day cycle. The treatment started three months post-HSCT (in median at day +96) until 1 year. Eighteen patients also received DLIs (in median 2/patient). Two-year CIR was 20% [188].

Recently the Dutch–Belgian Haemato-Oncology Foundation for Adults (HOVON) published the results of a phase I/II trial in high-risk AML or MDS. The patients underwent HSCT with RIC conditioning followed by maintenance with panobinostat (PAN) alone or combined with decitabine (DAC). In the first part of the study, three schedules were evaluated: PAN alone or PAN + DAC 10 or PAN + DAC 20. PAN was administered at a dose of 20 mg on days +1, +4, +8 and +11 of each cycle and DAC at 10 mg/m^2^ or 20 mg/m^2^ for 3 days in a 28-day cycles. The maintenance started on day 28. The cycles of epigenetic therapy were 4 and two DLIs were scheduled respectively after the second and fourth cycle. The combination PAN/DAC 20 encountered the DLT and was considered not feasible. The two-year CIR was 35%. PFS and OS at 2 years were, respectively 49% and 50% [189]. 

### 8.3. IDH-Inhibitors

Hot-spot mutations of the catalytic domain of the enzyme isocytrate dehydrogenase 1 (Arg 132) and 2 (arg 172–Arg 140) occur in approximately 10% of AML. Such mutations can induce the arrest of myeloid differentiation. IDH1 and IDH2 mutated genes can be the subjects of targeted therapy. Enasidenib (AG-221) and ivosidenib (AC120) are developed, respectively, as IDH-2 and IDH-1 inhibitors. Enasidenib, in relapsed/refractory IDH-2 mutated AML patients, produced an overall response rate of 26.6% [190]. 

The IDH-inhibitors are under investigation in relapsed/refractory AML and in untreated AML or MDS. 

The phase Ib/II trial NCT 04774393 studies the combinatory effect of decitabine/Cedurizine (ASTX727) and venetoclax with ivosidenib (arm A) or Enasidenib (arm B) in relapsed/refractory AML. The primary end-point for phase Ib is the dose-limiting toxicity (DLT) and for phase II the ORR. 

The NCT 03839771 study is a phase III, randomized, placebo-controlled study on ivosidenib or enasidenib combined with induction and consolidation therapy in newly diagnosed AML or MDS/AREB 2 harboring IDH-mutations. The primary end-point is EFS. 

The NCT02677922 trial is a phase Ib/II study investigating the efficacy and safety of ivosidenib or enasidenib combined with azacytidine in untreated, IDH-mutated AML considered ineligible for intensive chemotherapy. The primary end-points are DLT for phase Ib and ORR for phase II.

## 9. Extramedullary Relapse of AML

The extramedullary relapse (EMR) of AML after HSCT is a rare condition. The incidence reported in the literature varies between 2% and 5% [191,192,193,194]. The most common sites of involvement are the central nervous system (CNS), skin, skeleton, breast, testis and more rarely, muscle, serous membranes and mediastinum [193,194,195]. The risk factors for EMR are pre-transplant extramedullary involvement [191,192,193,194], monoblastic leukemia, expression of CD56+, 11q23 abnormalities [196,197] and hyperleukocytosis at diagnosis [191,192,193,194]. With regard to the SNC relapse, the retrospective analysis reported an incidence varying between 2% and 4% after HSCT and, as risk factors the previous SNC involvement, hyperleukocytosis at diagnosis [191], FLT3-ITD mutation and LDH > 1000 U/L at diagnosis [192]. The treatment strategies for EMR vary from surgical excision to chemotherapy and radiotherapy, according to the clinical presentation [198,199,200]. With regard to the SNC relapse, the conventional options include triple intratecal (TIT) chemotherapy (cytarabine 50–75 mg, methotrexate 12 mg and dexametasone 5 mg), CNS irradiation (CNSI) and high-dose cytarabine (HD ARA-C: 1.5–2 g/m^2^ bid for 3 days) [194,201,202]. The outcome also varies according to the number of lesions and the sites of involvement. The retrospective analysis based on heterogeneous EMR, including SNC, skin and other soft tissues, reported a survival not inferior than that of BM relapse after HSCT (8–10). Min Shi and co-workers in a retrospective case series including both ALL and AML, reported a median survival for EMR superior to BM relapse (respectively, 18 months vs. 10 months; *p* = 0.0001) [199]. Similarly, authors from the University of Minnesota reported a better outcome for the isolated EMR of AML than BM relapse [198]. A Japanese cooperative group reported a 1-year OS rate of 38% and 16%, respectively for EMR and BM relapse, without significant difference (*p* = 0.27). The survival after SNC relapse of AML varies from 0 to 26%, according to the period of observation, the age of the patients and the treatment employed. In any case, the SNC relapse of AML significantly reduces the life expectancy of the transplant recipients [191,194,196]. Treatment of SNC relapse is difficult both for the “sanctuary” characteristics of the nervous system and for the poor medical fitness of the patient who is not always eligible for intensive chemotherapy. The most appropriate intervention of SNC relapse should be tailored to the patient and to the clinical presentation. The first intervention for the leptomeningeal involvement with blast cells in cerebrospinal fluid (CSF) is the TIT administered biweekly until CNS complete remission and followed by a “consolidation” every 28–30 days up to at least five administrations. CNS complete remission is defined as a complete blasts disappearance from CSF in at least two consecutive lumbar punctures, complete resolution of neurological symptoms and of neuro-radiological lesions by CT and/or MR [202]. In case of greater severity of the clinical presentation at relapse (e.g., cranial nerve palsy, spinal roots nerve symptoms, intracranial hypertension or mass lesions by CT/MR) as well as in case of refractoriness to TIT, we suggest using HD ARA-C if the patient is eligible to intensive chemotherapy. CNSI, according to the previous patient’s radiation dose, might be useful if the disease is refractory to chemotherapy, in case of relapse after chemotherapy or if the patient is not eligible for intensive chemotherapy. To date, the use of new drugs in this setting appears far from clinical practice. A depot formulation of lyposomal cytarabine, that had been developed in the previous 15 years [203] at a dosage of 50 mg was associated with significant neurotoxicity compared to conventional IT in a randomized study in ALL patients [204].

## 10. Concluding Remarks

Allogeneic hematopoietic stem cell transplantation, in eligible patients, is the only curative and potentially healing approach. However, the relapse rate post-HSCT is still high, being around 40 or 50% and in certain subsets of patients (e.g., TP53-mutated, poor-risk cytogenetics) rising up to 70–80% [4,205,206,207].

It should be pointed out that the best prophylaxis for post-transplant relapse starts from the pre-transplant treatment. It is well-known that achieving complete remission with the absence of minimal-residual disease prior to HSCT provides the best chance of cure [208]. New knowledge in the field of minimal-residual disease, identification of driver mutations involved in the pathogenesis of disease, development of new targeted agents and their introduction in the clinical practice in combination with conventional chemotherapy have improved the outcome of AML. This new knowledge makes it possible to tailor the therapy to the patient.

The strict monitoring of MRD after transplantation is crucial in order to start a prompt intervention based on cellular and pharmacological strategies, and to prevent the overt relapse.

Novel targeted agents may be employed in the post-transplant maintenance and some of them have yet provided evidence for their effectiveness from randomized studies while others are actually the subject of controlled, prospective trials. Moreover, a better understanding of the immunological mechanisms underlying relapse extends the clinical armamentarium suitable for the management of the leukemia relapse to the immunological therapies. In certain clinical situations the new agents (HMAs, FLT3-inhibitors) might be used in combination with conventional strategies such as DLIs.

The SNC-relapse still remains a challenging situation that adversely affects the outcome of the patient.

The decision to perform a second transplantation should be made with great caution, after a careful evaluation of the risk–benefit ratio. It is not possible to give a precise indiation in this regard and the decision must be made on the basis of the particular clinical case.

Unlike what has been developed for acute lymphoblastic leukemia, further away from the clinical practice appears, for the moment, the use of the new cellular therapies in AML.

Last, but not least, it should be emphasized that every decision should be carefully discussed with the patient whose role in the decision-making process is to be considered central, without neglecting, also the emotional, social, logistical and financial aspects.

## Figures and Tables

**Table 1:** HMAs in the treatment of overt relapse: clinical results from retrospective and prospective trials. Abbreviations: CR: complete remission, PR: partial remission, CRi: complete remission with incomplete hematologic recovery, MLFR: myeloid leukemia free state, no resp: no response, SD: stable disease, CIR: cumulative incidence of relapse, ORR: overall response rate, OS: overall survival, RFS: relapse-free survival, EFS: event-free survival, AML: acute myeloid leukemia, MDS: myelodysplastic syndrome, AZA: azacitidine, DAC: decitabine, mo: months, na: not applicable. DLI: donor lymphocyte infusion, HMAs: hypomethylating agents, CHT: chemotherapy.

Authors	Number of Patients	Study Design	Schedule of Administration	Response	*p*-Value	Outcome	*p*-Value
Craddock et al., 2016 [78]	181 (116 AML, 65 MDS)	Retrospective	AZA 75 mg/m^2^ for 5–7 days	CR: 8% > 2 risk factors; CR: 34% no risk factors	*p* < 0.0019	2-year OS: 3% > 2 risk factors; 37% no risk factors	*p* < 0.00001
Motabi I et al., 2016 [79]	100 (CHT: 73 vs HMAs: 27)	Retrospective	AZA 75 mg/m^2^ for 5 days + DLI (55% and 33% of pts, respectively)	CR/CRi 51% vs. 19%	*p* < 0.004	Median PFS:4.9 vs. 3.8 months In favour of CHT	*p* < 0.02
Schroeder et al., 2018 [80]	36 (29 AML, 7 MDS)	Retrospective	DAC 20 mg/m^2^ for 5 days (67%) or 10 days (33%)	ORR: 25% CR: 17% PR: 8%	na	2-year OS: 11% (±6%)	na
Schroeder et al., 2013 [84]	30 (28 AMl, 2 MDS)	Prospective, Open Label, Single-Arm, Phase II	AZA 100 mg/m^2^ for 5 days + DLI	ORR: 30% CR: 23% PR: 7%	na	Median OS 117 mo (not reached for pts in CR, 83 days for pts without CR)	*p* < 0.001 in favour of patients achieving CR
Ghobadi A. et al., 2016 [79]	8 AML	Prospective, Open Label, 3 + 3 dose escalation, Phase I	AZA 45 mg/m^2^ (37%) and 75 mg/m^2^ (63%) at days 4-6-8-10 after DLI	ORR: 75% CR/CRi: 50% Cytogenetic remission: 25%	na	Median DFS: 2.9 mo Median OS: 12.5 mo	na
Craddock C. et al., 2019 [86]	29 (24 AML, 5 MDS)	Prospective, Open Label, Dose finding, Phase I	AZA 75 mg/m^2^ for 7 days + Lenalidomide (4 dose-levels: 5-10-15-25 mg)	ORR: 47% CR/CRi: 40% PR: 6%	na	Median OS: 10 mo for not-responders, 27 mo for responders	*p* = 0.004 in favour of responders
Woo J. et al., 2017 [87]	39 (26 AML, 13 MDS)	Prospective Open-label, Single-Arm, Phase II	AZA 75 mg/m^2^ for 7 days	ORR: 30.7% CR: 8% PR: 23%	na	2-year OS: 25% 71 % in responders)	na
Zhao P. et al., 2021 [90]	26 AML	Prospective Open-label, Single-Arm, Phase II	AZA 75 mg/m^2^ for 5 days + Venetoclax followed by DLI	ORR: 61.5% CR: 26.9% PR: 34.6%	na	Median EFS: 120 days Median OS: 284.5 days	na

**Table 2:** Studies on second HSCT. Abbreviations: HSCT: hematopoietic stem cell transplantation, CR: complete remission, PR: partial remission, CRi: complete remission with incomplete hematologic recovery, MLFR: myeloid leukemia free state, no resp: no response, SD: stable disease, CIR: cumulative incidence of relapse, ORR: overall response rate, OS: overall survival, RFS: relapse-free survival, EFES: event-free survival, DFS: disease-free survival, AML: acute myeloid leukemia, MDS: myelodysplastic syndrome, ALL: acute lymphoblastic leukemia, mts: months, DLI: donor lymphocyte infusion, MRD: matched-related donor, UD: unrelated donor, MMRD: mismatched-related donor, MMUD: mismatched unrelated donor, Haplo: haploidentical, EBMT: European bone marrow transplantation, CIBMTR: Center International for Blood and Marrow Transplantation Research, MDACC: MD Anderson Cancer Center, GETH: Group Espanol de Transplante Hematopoyetico.

Authors	Register/ Institute	N Pts	Period	Donor Type (MRD, MMRD, UD, MMUD, HAPLO)	Outcome (OS, LFS)	Risk Factors (*p*-Value)
Christopeit M. et al., 2013 [91]	EBMT	179 (132 AML, 46 ALL, 1 unclassifiable leukemia)	1998–2009	Previous MRD: Same MRD: 51% Different MRD: 11% UD: 38% Previous UD: Same UD (42%) Different UD (58%)	2-year LFS: 21% 2-year OS: 25%	OS advantage: -Previous CR longer than 6 mts (*p* < 0.001) -CR at 2nd HSCT (*p =* 0.006) -1st HSCT from MRD than UD (*p* = 0.042)
Orti G. et al., 2016 [92]	GETH	116 (88 AML, 25 MDS, 3 MPN)	1979–2011	MRD: 83% UD: 11% MMRD: 4% Syngenic: 2%	5-year DFS: 30% 5-year CIR: 37.8% 5-year OS: 32%	Shorter OS: -active disease (*p* < 0.001.) -time between 1st and 2nd HSCT <430 days (*p* < 0.001). -2nd HSCT from a donor different from MRD. (*p* = 0.03)
Shimoni A. et al., 2019 [96]	EBMT	556 AML	2006–2016	3 groups: 1- From 1st MRD or UD to 2nd same donor group 2- From 1st MRD or UD to 2nd different MRD or UD 3- From 1st MRD or UD to 2nd HAPLO	2-year LFS: 23.5% vs. 23.7% vs. 21.6% (*p* = 0.3) 2-year CIR: 51% vs. 49% vs. 44% (*p* = 0.9) 2-year OS: 36.4% vs. 28.7% vs. 23.3% (*p* = 0.21)	Shorter LFS and OS: -relapse <6 mo after 1st HSCT (*p* = 0.01) -advanced age (*p* = 0.07). -2nd HAPLO donor (*p* = 0.07). Better LFS and OS: -CR at 2nd HSCT (*p* = 0.002)
Kharfan-Dabaja M. et al. [97] 2021	EBMT	455 AML	2006–2019	2nd HSCT from UD (320 pts) vs. Haplo (135 pts)	2-year LFS: 25% vs. 29% (*p* = 0.73) 2-year OS: 31% vs. 29% (*p* = 0.57)	OS advantage: -Relapse > 1 year after 1st HSCT (*p* < 0.0001) -CR at 2nd HSCT (*p* = 0.02)
Rank A. et al., 2021 [98]	EBMT	45 (34 AML, 11 ALL)	2001–2018	3rd HSCT: 25 pts different donor from 2nd HSCT; 30% of pts had at least 2 donors	1-year PFS: 11% 1-year OS: 20%	OS/PFS advantage: -Change donor at least one time (*p* = 0.009/*p* = 0.007) -KS > 80% (*p* = 0.083/*p* = 0.046) -3rd HSCT from UD (*p* = 0.014/*p* = 0.012) -3rd HSCT after 2010 (*p* = 0.011/*p* = 0.012)
Kharfan-Dabaja M. et al. [99] 2018	EBMT	418 AML	1992–2015	Retrospective comparison 2nd HSCT vs. DLIs2 groups: 2nd HSCT (MRD 56%, UD 43%) vs. DLIs (MRD 54%, UD 46%)	2-year OS in pts relapsing <6 mo: 11% vs. 9% (*p* = 0.86) 2-year OS in pts relapsing >6 mo: 36% vs. 37% (*p* = 0.53) 2-year OS in pts in CR at intervention: 35% vs. 51% (*p* = 0.22) 2-year OS in pts not in CR at intervention: 20% vs. 19% (*p* = 0.59)	OS advantage: -Relapse > 6 months after 1st HSCT (*p* < 0.001) -CR at 2nd HSCT (*p* = 0.001) -prior cGVHD (*p* = 0.02)
Yalniz et al., 2021 [94]	MDACC	91 AML	2000–2019	MRD: 41% UD: 37% HAPLO: 21% Cord Blood: 1%	2-year PFS: 27% 2-year OS: 36%	Shorter OS: -cGVHD after 1st HSCT (*p* = 0.001) -HCT-CI ≥2 at 2nd HSCT (*p* < 0.003). -Relapse < 6 months after 1st HSCT (*p* < 0.02) -2nd HSCT before 2011 (*p* = 0.02) Shorter PFS: -cGVHD after 1st HSCT (*p* = 0.001) -HCT-CI ≥2 at 2nd HSCT (*p* = 0.01)
Duncan CN et al., 2015 [93]	CIBMTR	146 Children (64 AML, 66 ALL, 12 MDS, 4 JMMS) 179 Adults (111 AML, 54 ALL, 14 MDS)	1980–2009	Children: MRD: 53% UD: 41% Other Related: 5% Adults: MRD: 55% UD: 40% Other Related: 4%	2-year OS: Children: 83% Adults: 75% 6-year OS: Children: 64% Adults: 51% 10-year OS: Children: 55% Adults: 39%	Shorter OS: -Disease not in CR before 2nd HSCT (<0.01)

**Table 3:** HMAs in the maintenance therapy: clinical results from prospective trials. Abbreviations: CR: complete remission, PR: partial remission, CRi: complete remission with incomplete hematologic recovery, MLFR: myeloid leukemia free state, no resp: no response, SD: stable disease, CIR: cumulative incidence of relapse, ORR: overall response rate, OS: overall survival, RFS: relapse-free survival, EFS: event-free survival, AML: acute myeloid leukemia, MDS: myelodysplastic syndrome, AZA: azacitidine, DAC: decitabine, mo: months, na: not applicable, DLI: donor lymphocyte infusion, rh-G-CSF: recombinant-human granulocyte colonies-stimulating factor.

Authors	Number of Patients	Study Design	Schedule of Administration	Relapse	Outcome
De Lima et al., 2010 [113]	45 (37 AML, 8 MDS)	Open Label, dose escalation, Phase I	Optimal: AZA 32 mg/m^2^ dd 1–5 25-day cycle	20 mts follow-up CIR: 53%	1-year EFS: 55% 1-year OS: 77%
Pusic I et al., 2015 [114]	22 evaluable (17 AML, 5 MDS)	Open Label, dose escalation, Phase I	Optimal: DAC 10 mg/m^2^ dd 1–5 6-wks cycle	2-year CIR: 28%	2-year DFS: 48% 2-year OS: 56%
Craddock C. et al., 2016 [78]	37 AML	Open Label, Single-Arm, Phase II	AZA 36 mg/m^2^ days 1–5	Median time to relapse: 8 months	2-year RFS: 49% 2-year OS: 49%
De Lima et al., 2018 [116]	30 (26 AML, 4 MDS)	Open Label, 3+3 dose escalation, Phase I/II	Oral AZA. 4 dose-levels: 200 mg for 7 days 300 mg for 7 days 150 mg for 14 days 200 mg for 14 days	1-year CIR: 43% 7-days group; 13% 14-days group	Median OS: not reached. Estimated 1-year OS: 81% for 7-day group, 86% for 14-day group
Guillaume T. et al., 2019 [117]	30 (20 AML, 10 MDS)	Open Label, Single-Arm (compared with historical cohort not receiving AZA or DLIs), Phase II	AZA 32 mg/m^2^ days 1–5 + DLI	2-year CIR: 27%. (41% in historical cohort, *p* = 0.2)	2-year DFS: 65.5% 2-year OS: 65.5%
Oran B. et al., 2020 [118]	187 (140 AML, 47 MDS)	Randomized 1:1, Open Label, Double Arm, Phase III	AZA 32 mg/m^2^ days 1–5 vs. observation only	1-year CIR: 41% vs. 39%*p* = ns	RFS: 2.07 y vs. 1.28 y (p = ns) OS: 2.52 y vs. 2.56 y (p = ns)
Lei Gao et al., 2020 [120]	204 AML	Randomized 1:1, Open Label, Double Arm, Phase III	rhG-CSF 100 µg/m^2^ days 0–5 + DAC 5 mg/m^2^ days 1–5 vs. observation only	2-year CIR: 38% vs. 15% (*p* < 0.01)	2-year LFS: 81.9% vs. 60.7% 2-year OS: 85.8% vs. 69.7%

**Table 4:** Results of trials with CAR-T post-HSCT. Abbreviations: CR: complete remission, PR: partial remission, CRi: complete remission with incomplete hematologic recovery, MLFR: myeloid leukemia free state, no resp: no response, SD: stable disease.

Authors	Target	CAR Construct	*n*. pts	Best Response
Ritchie, 2013 [154]	CD33	CD28-CD3	4	1 CR 1 PR
Wang, 2015 [155]	CD 33	4-1BB	1	1 PR
Tang, 2018 [156]	CD 33	CD28-4-1BB	3	1 CR
Yao, 2019 [157]	CD 123	4-1BB	1	1 CRi
Cummins, 2019 [158]	CD 123	CD28-CD3		1 CR
Zhang, 2021 [152]	CCL-1	NR	3	3 CR
Baumeister, 2013 [159]	NKG2D-L	CD3	7	7 No resp
Sallman, 2020 [160]	NKG2D-L	CD3	22	1 MLFR 1 PR 6 SD 14 no resp
Danylesko, 2020 [161]	CD19	CD28	1	1 CR
Liu F, 2021 [EHA 2020] [162]	CLL-1–CD33	NR	9	7 CR
Tambaro, 2021 [163]	CD33	4-1BB	10	No resp
Budde LE, 2019 (EHA CAR-T 2019) [164]	CD123	4-1BB mRNA vector	5	5 No resp
Deeren, 2021 [ASH 2020] [165]	NKG2D-L	CD3	2	1 SD 1 no resp
Total			75	17 CR/CRi/CRh

## Data Availability

Not applicable.
